# Arboviruses antagonize insect Toll antiviral immune signaling to facilitate the coexistence of viruses with their vectors

**DOI:** 10.1371/journal.ppat.1012318

**Published:** 2024-06-12

**Authors:** Dongsheng Jia, Guozhong Luo, Heran Guan, Tingting Yu, Xinyan Sun, Yu Du, Yiheng Wang, Hongyan Chen, Taiyun Wei

**Affiliations:** State Key Laboratory of Ecological Pest Control for Fujian and Taiwan Crops, Vector-borne Virus Research Center, Fujian Agriculture and Forestry University, Fuzhou, Fujian, China; University of California, Davis Genome Center, UNITED STATES

## Abstract

Many plant arboviruses are persistently transmitted by piercing-sucking insect vectors. However, it remains largely unknown how conserved insect Toll immune response exerts antiviral activity and how plant viruses antagonize it to facilitate persistent viral transmission. Here, we discover that southern rice black-streaked dwarf virus (SRBSDV), a devastating planthopper-transmitted rice reovirus, activates the upstream Toll receptors expression but suppresses the downstream MyD88-Dorsal-defensin cascade, resulting in the attenuation of insect Toll immune response. Toll pathway-induced the small antibacterial peptide defensin directly interacts with viral major outer capsid protein P10 and thus binds to viral particles, finally blocking effective viral infection in planthopper vector. Furthermore, viral tubular protein P7-1 directly interacts with and promotes RING E3 ubiquitin ligase-mediated ubiquitinated degradation of Toll pathway adaptor protein MyD88 through the 26 proteasome pathway, finally suppressing antiviral defensin production. This virus-mediated attenuation of Toll antiviral immune response to express antiviral defensin ensures persistent virus infection without causing evident fitness costs for the insects. E3 ubiquitin ligase also is directly involved in the assembly of virus-induced tubules constructed by P7-1 to facilitate viral spread in planthopper vector, thereby acting as a pro-viral factor. Together, we uncover a previously unknown mechanism used by plant arboviruses to suppress Toll immune response through the ubiquitinated degradation of the conserved adaptor protein MyD88, thereby facilitating the coexistence of arboviruses with their vectors in nature.

## Author summary

Many devastating plant viral pathogens are persistently transmitted by arthropod insects without causing evident insect fitness costs. However, how Toll pathway plays an antiviral role and how viruses antagonize Toll antiviral immune response are still poorly understood. Here, we report that the planthopper defensin, a type of antibacterial peptide induced by Toll pathway, directly binds to viral particles of an important rice reovirus, thus exerting antiviral activity. However, the virus-encoded protein promotes the ubiquitinated degradation of the conserved Toll pathway adaptor protein MyD88, finally suppressing defensin production. Such process finally ensures persistent virus transmission by insect vectors. Our findings provide insights into how viruses have evolved the novel strategies to evade and even exploit insect Toll antiviral immune response to facilitate persistent viral transmission.

## Introduction

Many arthropod-borne viruses (arboviruses) have a significant impact on agriculture and human health and are persistently transmitted by insect vectors [1]. For instance, rice stripe virus (RSV), transmitted by planthoppers, poses a serious agricultural threat in rice-growing countries across Asia, while mosquito-transmitted Zika virus poses a significant threat to public health worldwide [2,3]. Importantly, persistent viral transmission does not seem to cause any noticeable negative effects on insect fitness, suggesting that insect vectors have developed immune tolerance mechanisms to ensure the continuous infection of arboviruses in nature [4,5]. During the persistent viral transmission by insect vectors, viruses initially infect the insect intestines, then spread to the hemolymph, and eventually enter the salivary glands, from where they are released to susceptible hosts [6]. Generally, the virus encounters various antiviral immune pathways within the vector, including melanization, autophagy, apoptosis, small interfering RNA (siRNA), Toll, immunodeficiency (IMD), and JAK/STAT [7–10]. However, the viruses have evolved several strategies to evade and even exploit the antiviral immune pathways of insect vectors to facilitate persistent viral transmission [11,12]. Developing a deeper understanding of the balanced interplay among viral transmission, insect fitness, and innate antiviral immunity would aid in illustrating how arboviruses adapt and coexist with their vectors.

The Toll pathway is a critical innate immune signaling system that plays a role in the antiviral defense of insects by regulating the production of effector molecules [13]. Within the insect Toll signaling pathway, the transmembrane protein spaetazle (Spz) binds to Toll receptors, and the myeloid differentiation factor 88 homologue (MyD88) recruits Tube and Pelle to form a complex [14]. This complex then initiates the translocation of the NF-κB family member transcription factor Dorsal from the cytoplasm to the nucleus, where it regulates the expression of various antibacterial peptides (AMPs) [15]. AMPs, including diptericin, cecropin, cathelicidin, and defensin, play a critical role in the insect innate immune system due to their broad-spectrum antibacterial and antiviral activities [16]. Defensins, a type of small AMP, are specifically produced by insects as part of their innate immune defense and are characterized by their highly conserved cysteine-rich structure, which is essential for their antimicrobial activity [17]. MyD88 serves as a crucial adaptor protein within the Toll pathway, connecting the receptors to downstream signaling pathway components [18–20]. The significance of the Toll pathway in combating viruses was first reported in *Drosophila* infected with Drosophila X virus [21]. In both *Drosophila* and mosquitoes, the Toll pathway plays a role in resistance against various RNA viruses, such as Drosophila C virus, cricket paralysis virus, flock house virus, and dengue virus [7,13,14,22,23]. Many plant arboviruses are persistently transmitted by planthoppers, leafhoppers, aphids, whiteflies, and thrips [1]. The Toll pathway also plays an antiviral role during RSV infection in planthopper vector [8]. Recent report shows that RSV-encoded nonstructural protein NS4 could antagonize planthopper vector antiviral Toll immune response through competitively binding to the transcription factor Dorsal that mediates the downstream antiviral response [24]. However, how plant arboviruses antagonize Toll immune response to induce antiviral AMPs to ensure persistent virus transmission by insect vectors is still unknown.

In recent years, planthopper- and leafhopper-borne rice viruses have spread rapidly throughout southern China and Southeast Asia [25,26]. The white-backed planthopper *Sogatella furcifera*, in particular, has been responsible for the transmission of the reovirus southern rice black-streaked dwarf virus (SRBSDV), leading to epidemic outbreaks and significant losses in rice yield over the past two decades [27]. When rice viruses persistently infect insect vectors, it induces immune homeostasis to regulate the coexistence of the vector and the virus. SRBSDV triggers a conserved siRNA antiviral pathway to control excessive viral accumulation in insect vectors and ensure optimal replication [9,28]. Additionally, the nonstructural protein P7-1 of SRBSDV assembles tubular or fibrillar structures that enable efficient viral spread in insect vectors [27,29,30]. These fibrillar structures target mitochondria directly and cause mitochondrial degeneration [30]. The degenerated mitochondria are then recruited into P7-1-induced autophagosomes to initiate mitophagy, preventing mitochondria-dependent apoptosis and promoting persistent viral propagation in insect vectors [31]. However, it is still unclear whether other immune pathways play a role in protecting insect vectors from SRBSDV infection.

In this study, we reveal that the infection of *S*. *furcifera* by SRBSDV activates the upstream Toll receptors expression but suppresses the downstream MyD88-Dorsal-defensin cascade, thereby antagonizing the Toll antiviral immune response. Defensin, a type of antibacterial peptide induced by Toll pathway, directly binds to viral particles, thus exerting antiviral activity. SRBSDV P7-1 directly binds to and promotes E3 ubiquitin ligase-mediated ubiquitinated degradation of MyD88. This finding reveals a new mechanism exploited by arboviruses to suppress the Toll immune response through the ubiquitinated degradation of the conserved adaptor protein MyD88, facilitating persistent viral infection in insect vectors without apparent fitness cost. Our results uncover the dual regulation of mitophagy and the Toll pathway by SRBSDV P7-1, contributing to the understanding of the homeostasis between vectors and arboviruses.

## Results

### SRBSDV antagonizes planthopper Toll antiviral pathway by suppressing the downstream MyD88-Dorsal-defensin cascade

We investigated the expression of the Toll signaling cascade (Toll receptors-MyD88-Dorsal-defensin) upon SRBSDV infection in planthopper *S*. *furcifera*. Previous transcriptome analysis showed that *S*. *furcifera* possessed the conserved Toll signaling cascade mainly involving 5 Toll receptors (Toll6, Toll7, Toll8, Toll10, and Toll13), the adaptor protein MyD88, and the downstream transcription factor Dorsal (S1A Fig) [32]. Furthermore, *S*. *furcifera* lacked the genes encoding many known insect AMPs, such as diptericin, attacins, drosocin, cecropin, and drosomycins (S1A Fig) [32]. However, it possessed a defensin protein with a DEFL defensin-like domain at its C-terminus (S1B Fig).

**Fig 1:**
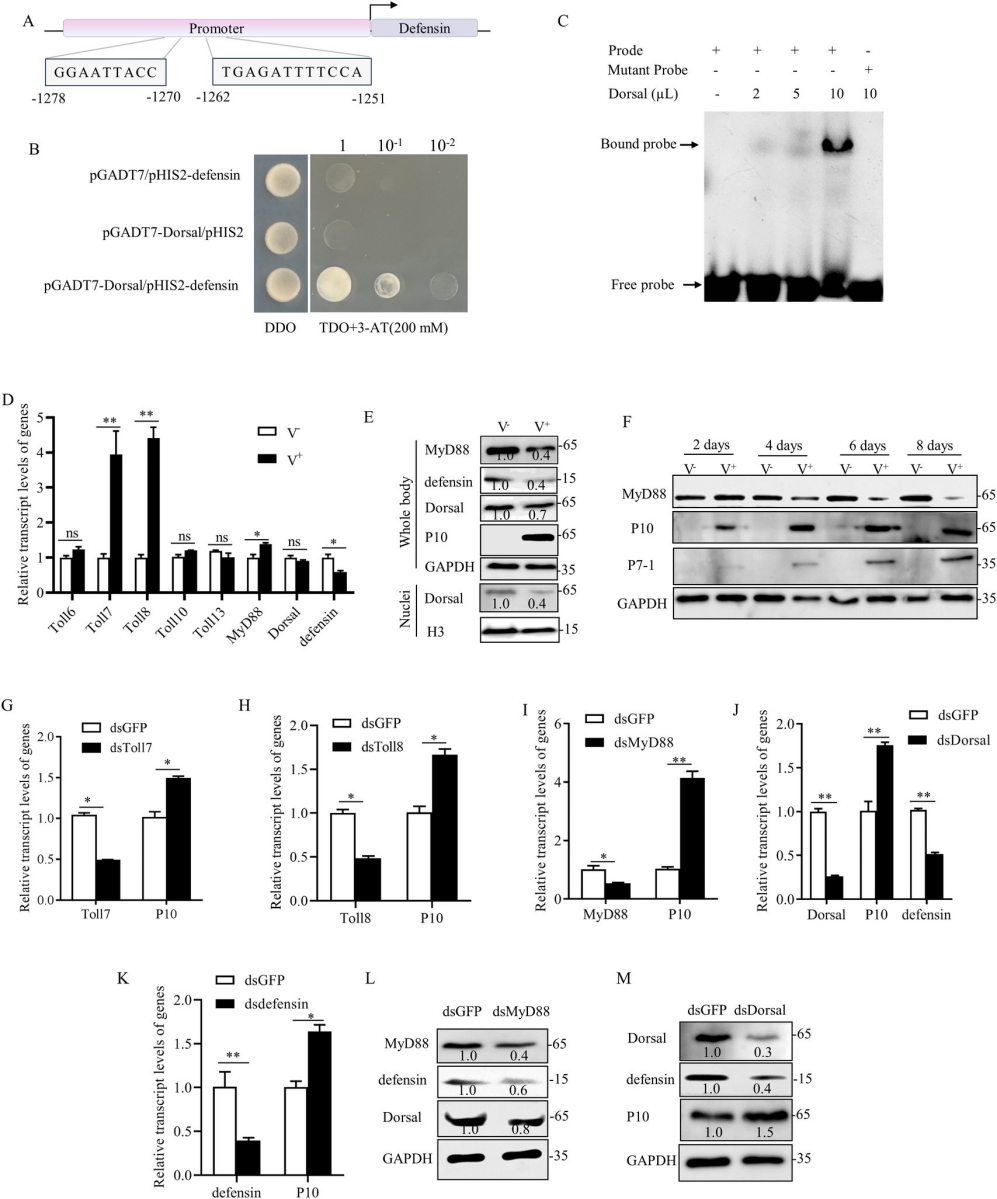
Effects of SRBSDV infection on the Toll antiviral signaling cascade in *S*. *furcifera*. (A) The sites of potential Dorsal-binding motifs in the promoter region of *defensin*. (B) Verification for the binding of Dorsal to the promoter sequences of *defensin* using Y1H assay. The different combinations of constructs transformed into yeast cells were grown on DDO (SD/-Leu/-Trp) medium, and interactions were detected on TDO (SD/-Leu/-Trp/-His) media supplemented with 200 mM 3-AT. (C) Verification for the binding of Dorsal to the promoter sequences of *defensin* using EMSA with the *defensin*-Cy5-probe. The Cy5-labeled mutated probe was used as the negative control. (D) The relative transcript levels of *Toll6*, *Toll7*, *Toll8*, *Toll10*, *Toll13*, *MyD88*, *Dorsal*, and *defensin* in 30 nonviruliferous or viruliferous insects, as determined by RT-qPCR assays. (E) The accumulation levels of MyD88, defensin, Dorsal and P10 in 30 nonviruliferous and viruliferous insects, as determined by western blot assays. The accumulation level of Dorsal in cellular nuclei of 30 nonviruliferous and viruliferous insects was also shown. Insect GAPDH served as the reference of total proteins. Insect H3 served as the reference of nuclear proteins. (F) The accumulation levels of MyD88, P10 and P7-1 in 30 nonviruliferous and viruliferous insects at 2, 4, 6 and 8 days padp, as determined by western blot assays. (G-K) Effects of knocking down *Toll7*, *Toll8*, *MyD88*, *Dorsal*, and *defensin* expression on the relative transcript levels of *P10*, as determined by RT-qPCR assays. Effect of knocking down *Dorsal* expression on the relative transcript level of *defensin* was also shown in J. Thirty dsRNAs-treated viruliferous insects were examined. (L) Effects of knocking down *MyD88* expression on the accumulation of Dorsal and defensin in 30 dsRNAs-treated nonviruliferous insects, as determined by western blot assay. (M) Effects of knocking down *Dorsal* expression on the accumulation of defensin and P10 of SRBSDV in 30 dsRNAs-treated viruliferous insects, as determined by western blot assay. Means (± SD) in D and G-K were shown from three biological replicates. *, *P*<0.05; **, *P*<0.01; ns, not significant. Insect GAPDH in F, L and M served as the reference of total proteins. The relative intensities of bands of different proteins were determined using ImageJ. Data represent three biological replicates.

We then investigated whether the transcription factor Dorsal of Toll pathway could directly bind to the promoter sequences of *defensin* in *S*. *furcifera*. The 2,000 bp putative promoter sequences of *defensin* of *S*. *furcifera* were obtained from the NCBI. Two putative Dorsal-binding motifs of *defensin* promoter sequences were identified at positions -1278 to -1270 bp and -1262 to -1251 bp by analyzing JASPAR database (Fig 1A). *Defensin* promoter region containing putative Dorsal-binding motifs was cloned, and its binding ability to Dorsal was assessed through yeast one-hybrid (Y1H) and electrophoretic mobility shift assays (EMSA). Y1H assay revealed the interaction of Dorsal with the promoter region of *defensin* (Fig 1B). EMSA assay confirmed that Dorsal specifically bound to the probe containing putative Dorsal-binding motifs (Fig 1C). Potentially, the transcription factor Dorsal directly binds to *defensin* promoter to activate *defensin* transcription.

RT-qPCR assays showed that SRBSDV infection resulted in the elevated transcript levels of *Toll7*, *Toll8*, and *MyD88*, as well as the decreased transcript level of *defensin*, with no significant changes observed in *Toll6*, *Toll10*, *Toll13*, and *Dorsal* transcript levels in viruliferous insects at 6 days post-first access to diseased plants (padp) (Fig 1D). However, western blot assays showed that SRBSDV infection led to the reduced accumulation of MyD88, Dorsal, and defensin in viruliferous insects at 6 days padp (Fig 1E). Accordingly, Dorsal accumulation level in cellular nuclei of viruliferous insects was also decreased (Fig 1E). We also investigated the dynamic protein accumulation levels of MyD88 during SRBSDV infection in *S*. *furcifera*. The accumulation levels of MyD88 in viruliferous insects increased at 2 days padp and then decreased from 4 days padp (Fig 1F). Thus, SRBSDV infection activates the upstream Toll7 and Toll8 expression, but suppresses the downstream MyD88-Dorsal-defensin cascade, thereby inhibiting the Toll signaling pathway.

To explore the potential antiviral roles of the Toll pathway, third instar nymphs of *S*. *furcifera* were allowed to feed on SRBSDV-infected rice plants for 2 days, and then were microinjected with dsRNAs targeting *Toll7*, *Toll8*, *MyD88*, *Dorsal*, *defensin*, or *GFP*. RT-qPCR assays showed that knockdown of *Toll7*, *Toll8*, *MyD88*, *Dorsal*, or *defensin* expression significantly increased viral accumulation in viruliferous insects at 6 days padp (Fig 1G–1K). Potentially, SRBSDV infection activates the upstream Toll7 and Toll8 expression for stimulating the downstream Toll signaling cascade. Western blot assays confirmed that knockdown of *MyD88* expression decreased downstream Dorsal and defensin accumulation in nonviruliferous insects (Fig 1L). Meanwhile, RT-qPCR and western blot assays confirmed that knockdown of *Dorsal* expression significantly decreased *defensin* expression in viruliferous insects (Fig 1J and 1M). Thus, virus-mediated the reduced accumulation of MyD88 potentially suppresses the downstream signaling cascade, finally reducing the translocation of the downstream Dorsal into the nucleus for regulating *defensin* expression. These results collectively suggest that SRBSDV has evolved to attenuate the antiviral role of the Toll pathway to promote viral infection in insect vectors.

### SRBSDV antagonizes planthopper Toll antiviral pathway to facilitate persistent virus infection in insect vectors

**Fig 2:**
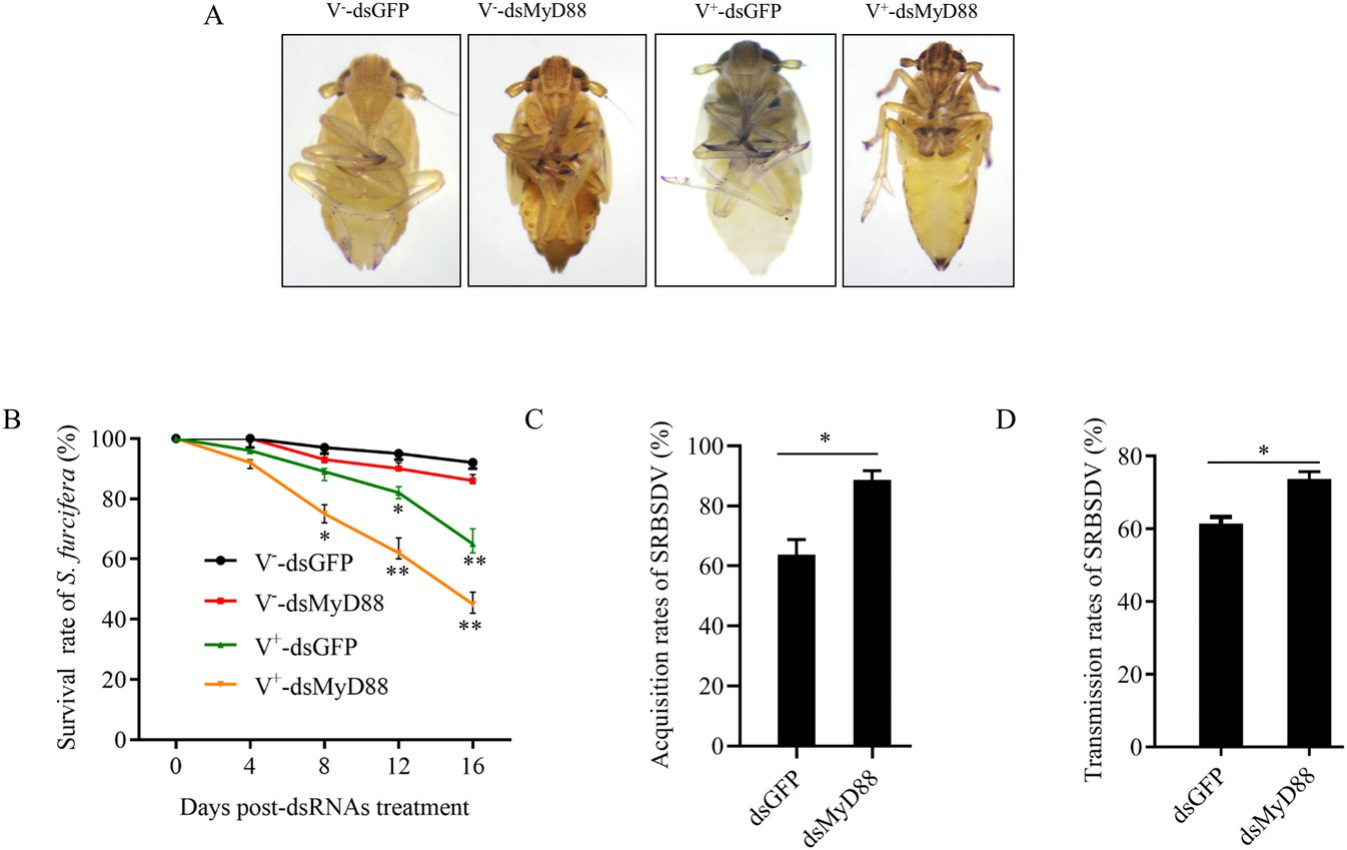
Effects of the suppression of Toll immune response on viral transmission efficiency and insect mortality. (A and B) Morphological characteristics (A) and survival rates (B) of 100 nonviruliferous or viruliferous insects treated with dsGFP or dsMyD88. V^—^dsGFP, dsGFP treated nonviruliferous insects; V^—^dsMyD88, dsMyD88 treated nonviruliferous insects; V^+^-dsGFP, dsGFP treated viruliferous insects; V^+^-dsMyD88, dsMyD88 treated viruliferous insects. (C) Acquisition rates of SRBSDV by 30 dsGFP- or dsMyD88-treated insects. (D) Transmission rates of SRBSDV by 30 dsGFP- or dsMyD88-treated viruliferous insects. Means (± SD) in B-D were shown from three biological replicates. *, *P*<0.05; **, *P*<0.01.

We proceeded to investigate whether the Toll pathway affected the fitness of *S*. *furcifera* during SRBSDV infection. Microinjection of dsRNAs targeting *MyD88* (dsMyD88) into third-instar nymphs of *S*. *furcifera* resulted in insignificant phenotypic abnormalities and death in nonviruliferous insects (Fig 2A and 2B). However, there was a higher mortality rate in dsMyD88-treated insects, compared to dsGFP-treated controls post viral infection (Fig 2A and 2B). Notably, approximately 30% of dsMyD88-treated viruliferous insects died, while only approximately 12% of dsGFP-treated viruliferous controls died at 8 days padp (Fig 2B). Thus, the significant increase in viral accumulation was associated with the increased mortality rate in dsMyD88-treated insects. In contrast, the significant increase in viral accumulation was associated with an increased mortality rate in dsGFP-treated insects starting from 12 days padp (Fig 2B). As expectedly, dsMyD88 treatment significantly increased the acquisition and transmission rates of SRBSDV by insect vectors (Fig 2C and 2D). Therefore, the further suppression of the Toll antiviral response through knockdown of *MyD88* expression in viruliferous *S*. *furcifera* led to the increased viral acquisition and transmission efficiency and insect mortality rate. These findings suggest that SRBSDV antagonizes Toll immune response to facilitate persistent virus infection in insect vectors.

### E3 ubiquitin ligase SfREL interacts with MyD88 and mediates its ubiquitinated degradation through the 26S proteasome pathway

**Fig 3:**
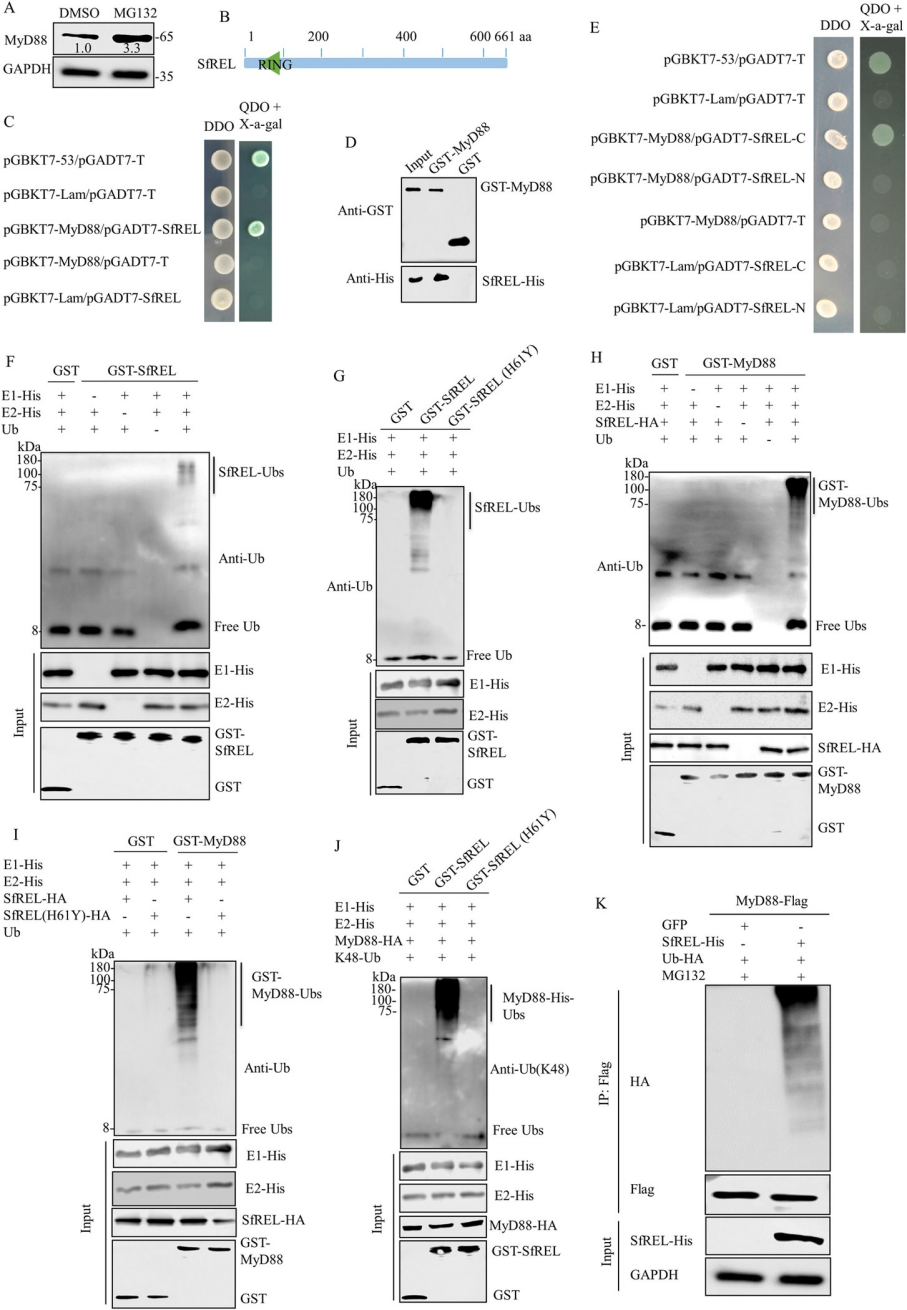
Interaction between MyD88 and SfREL *in vitro*. **(**A) The accumulation levels of MyD88 in viruliferous *S*. *furcifera* were increased in MG132 treatment. (B) Schematic representation of the RING domain of SfREL. (C) Interaction between MyD88 and SfREL in Y2H assays. Transformants were plated on DDO or QDO with X-α-gal. DDO, SD/-Trp-Leu medium. QDO, SD/-Trp-Leu-His-Ade medium. (D) Interaction between MyD88 and SfREL in GST pull-down assay. GST-MyD88 was incubated with glutathione-Sepharose beads. SfREL-His was then added to the beads, followed by western blot assay to detect SfREL-His bound to GST-MyD88. (E) Interaction between MyD88 and SfREL-N or SfREL-C in Y2H assays. F-I, *In vitro* ubiquitination assay of GST-SfREL or GST-MyD88 in the presence of E1 and E2, as determined by western blot assays. (F and G) Ubiquitination of GST-SfREL or GST-SfREL (H61Y) was analyzed using ubiquitin (Ub) antibody. (H and I) Ubiquitination of GST-MyD88 by SfREL-HA or SfREL (H61Y)-HA was analyzed using Ub antibody. (J) Ubiquitination of GST-MyD88 by SfREL-HA was analyzed using Ub (k48) antibody. (K) Immunoprecipitation analysis of the ubiquitination of MyD88 in Sf9 cells expressed with MyD88-Flag, Ub-HA, together with SfREL-HA or GFP. The input proteins were analyzed using His-Tag, GST-Tag, or HA-Tag antibody.

MyD88 is an essential adaptor protein in the Toll pathway, linking the receptors to downstream signaling pathway components [19]. At 6 days padp, SRBSDV infection significantly increased *MyD88* transcript level but decreased MyD88 protein expression level, suggesting post-translational degradation of MyD88 (Fig 1D and 1F). The 26S proteasome system is known to be one of the major pathways for protein degradation [33]. To confirm the role of the proteasome in SRBSDV-induced reduction of MyD88, we microinjected SRBSDV-infected *S*. *furcifera* with 10 μM MG132, the proteasome inhibitor, for 48 h. The results showed higher accumulation levels of MyD88 caused by MG132 treatment, suggesting that ubiquitinated degradation through the 26S proteasome pathway may be involved in SRBSDV-induced degradation of MyD88 (Fig 3A).

Ubiquitination is a cascade system involving three enzymes: E1 (ubiquitin-activating enzyme), E2 (ubiquitin-conjugating enzyme), and E3 (ubiquitin ligase), which leads to the covalent attachment of ubiquitin moieties to the target protein [33,34]. E3 ubiquitin ligases facilitate the attachment of lysine 48 (K48)-linked polyubiquitin chains to target proteins for subsequent proteasomal degradation [35]. In a previous study, we used MyD88 as a bait protein to screen for interactors from a cDNA library of *S*. *furcifera* using the yeast two-hybrid assay (Y2H). We identified a RING-type E3 ubiquitin ligase containing the RING finger domain in *S*. *furcifera* (SfREL) that interacted with MyD88. SfREL encodes a 661 amino acid protein that includes a canonical RING type (C3HC4) domain at its N-terminus (Figs 3B, S2A and S2B) and has been implicated in the ubiquitination-proteasome pathway [36,37]. Y2H and glutathione S-transferase (GST) pull-down assays confirmed the specific interaction between MyD88 and SfREL (Fig 3C and 3D). Furthermore, Y2H assay revealed that MyD88 specifically interacted with the C-terminal fragment of SfREL, but not with the RING domain-containing N-terminal fragment of SfREL (Fig 3E), indicating that SfREL may be involved in mediating the ubiquitinated degradation of MyD88.

To explore the relationship between SfREL and MyD88, an *in vitro* ubiquitination assay was conducted. The ubiquitinated form of SfREL was detected using the ubiquitin antibody in the presence of E1, E2, and ubiquitin, confirming that SfREL possesses E3 ubiquitin ligase activity (Fig 3F). However, an E3 ligase inactive mutant GST-SfREL (H61Y), in which His-61 in the C3HC4-type RING domain was replaced by Tyr, abolished the E3 ubiquitin ligase activity (Figs 3G and S2C). These results demonstrate that SfREL possesses E3 ligase activity, which is dependent on the presence of the C3HC4-type RING domain. Given that SfREL is an E3 ligase that interacts with MyD88, we then investigated whether SfREL can ubiquitinate MyD88. In the *in vitro* ubiquitination assays, GST-MyD88 was ubiquitinated by SfREL-HA in the presence of E1, E2, and ubiquitin (Figs 3H, 3I and S3). However, the ubiquitinated form of GST-MyD88 was not detectable when any of the proteins were removed from the reaction or GST-SfREL was replaced with the mutant GST-SfREL (H61Y) (Fig 3H and 3I). Furthermore, the form of SfREL-mediated MyD88 polyubiquitination was detected using the Ub-K48 antibody (Fig 3J). GST-MyD88 was ubiquitinated by GST-SfREL, but not by the mutant GST-SfREL (H61Y) or GST, in the presence of E1, E2, and ubiquitin (Fig 3J). Additionally, we investigated whether SfREL mediated the ubiquitinated degradation of MyD88 in Sf9 cells. Treatment with 50 μM MG132 significantly increased the accumulation of ubiquitinated MyD88-Flag in Sf9 cells co-expressing SfREL-His and MyD88-Flag but not in Sf9 cells co-expressing GFP and MyD88-Flag (Fig 3K). These findings demonstrate that MyD88 is a substrate of SfREL for ubiquitinated degradation *in vitro*.

### Viral nonstructural protein P7-1 promotes SfREL-mediated ubiquitinated degradation of MyD88

**Fig 4:**
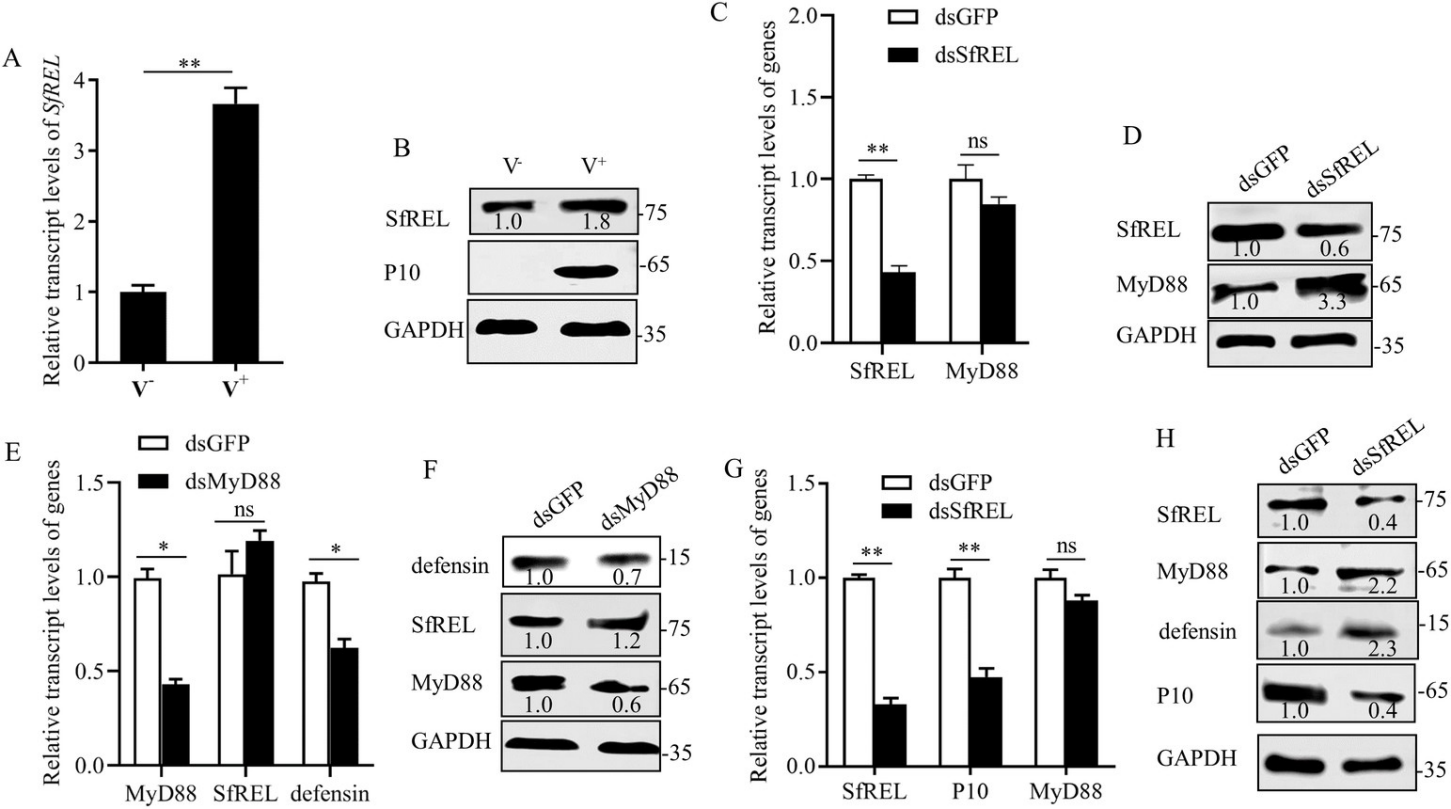
SfREL negatively regulates MyD88 expression to benefit viral infection in *S*. *furcifera*. **(**A and B) The relative transcript levels (A) and protein accumulation (B) of SfREL in 30 nonviruliferous and viruliferous insects, as determined by RT-qPCR and western blot assays. (C and D) The relative transcript (C) and protein accumulation (D) levels of MyD88 and SfREL in 30 dsGFP- or dsSfREL-treated nonviruliferous insects, as determined by RT-qPCR and western blot assays. (E and F) The relative transcript (E) and protein accumulation (F) levels of MyD88, defensin and SfREL in 30 dsGFP- or dsMyD88-treated nonviruliferous insects, as determined by RT-qPCR and western blot assays. (G and H) The relative transcript (G) and protein accumulation (H) levels of SfREL, P10, and MyD88 in 30 dsGFP- or dsSfREL-treated viruliferous insects, as determined by RT-qPCR and western blot assays. Means (± SD) in A, C, E and G were shown from three biological replicates. *, *P*<0.05; **, *P*<0.01; ns, not significant. Insect GAPDH in B, D, F and H was served as the reference protein. The relative intensities of bands of different proteins were determined using ImageJ. Data represent three biological replicates.

We then investigated whether SfREL mediated the downregulation of MyD88 in *S*. *furcifera*. RT-qPCR and western blot assays showed a significant increase in the expression of SfREL upon SRBSDV infection in insect vectors at 6 days padp (Fig 4A and 4B). This indicates that SRBSDV infection activates the expression of SfREL. Subsequently, dsRNAs targeting *SfREL* (dsSfREL) were microinjected into nonviruliferous nymphs of *S*. *furcifera* to knock down the expression of *SfREL*. The knockdown of *SfREL* increased the accumulation level of MyD88 but did not have a significant effect on the transcript level of *MyD88* (Fig 4C and 4D). Conversely, RT-qPCR and western blot assays showed that microinjecting nonviruliferous insects with dsMyD88 did not affect the transcript and protein accumulation levels of SfREL, but decreased the transcript and protein accumulation levels of defensin (Fig 4E and 4F). These findings suggest that SfREL directly binds to and negatively regulates the protein accumulation level of MyD88. Consistent with this, the knockdown of *SfREL* expression effectively increased the accumulation level of MyD88 but decreased viral accumulation in viruliferous insects (Fig 4G and 4H). Therefore, SRBSDV-activated SfREL promotes the ubiquitinated degradation of MyD88.

**Fig 5:**
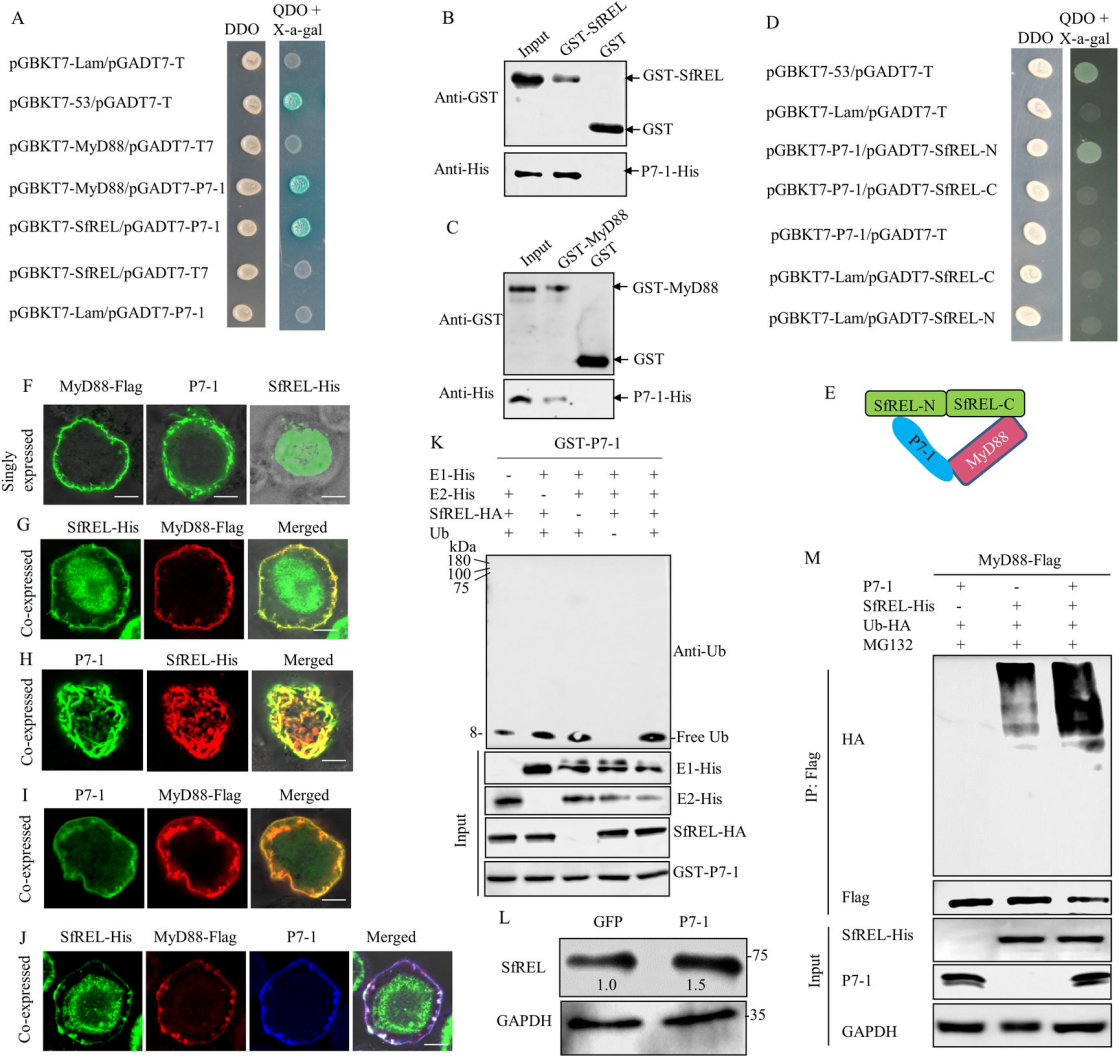
P7-1 interacts with SfREL and promotes SfREL-mediated ubiquitinated degradation of MyD88. (A) Interactions among P7-1, MyD88 and SfREL in Y2H assays. Transformants were plated on either DDO or QDO with X-α-gal. DDO, SD/-Trp-Leu medium. QDO, SD/-Trp-Leu-His-Ade medium. (B and C) Interaction between P7-1 and SfREL or MyD88 in GST pull-down assays. GST-SfREL or GST-MyD88 was incubated with glutathione-Sepharose beads. P7-1-His was then added to the beads, followed by western blot assay to detect P7-1-His bound to GST-SfREL or GST-MyD88. (D) Interaction between SRBSDV P7-1 and SfREL-N or SfREL-C in Y2H assays. (E) The interaction model among MyD88, SfREL and P7-1. (F-J) Colocalization among MyD88, SfREL, and P7-1 in Sf9 cells, as determined by immunofluorescence microscopy. (F) MyD88-Flag, P7-1, or SfREL-His singly expressed in Sf9 cells. Cells were respectively immunolabeled with Flag-Alexa Fluor 488 (green), P7-1-FITC (green), or His-Alexa Fluor 488 (green). The images were merged under a background of transmitted light. (G) Co-expression of SfREL-His and MyD88-Flag in Sf9 cells. Cells were immunolabeled with His-Alexa Fluor 488 (green) and Flag-Alexa Fluor 555 (red). (H) Co-expression of P7-1 and MyD88-Flag in Sf9 cells. Cells were immunolabeled with P7-1-FITC (green) and Flag-Alexa Fluor 555 (red). (I) Co-expression of P7-1 and SfREL-His in Sf9 cells. Cells were immunolabeled with P7-1-FITC (green) and Flag-Alexa Fluor 555 (red). (J) Co-expression of SfREL-His, MyD88-Flag, and P7-1 in Sf9 cells. Cells were immunolabeled with His-Alexa Fluor 488 (green), Flag-Alexa Fluor 555 (red), and P7-1-Alexa Fluor 647 (blue). The merged images in G-J were under a background of transmitted light. Bars, 5 μm. (K) *In vitro* ubiquitination of GST-P7-1 by SfREL-HA, as determined by western blot assays using ubiquitin (Ub) antibody. The input proteins were analyzed using His-Tag, GST-Tag, or HA-Tag antibodies. (L) The accumulation of SfREL in nonviruliferous insects, as determined by western blot assays. Thirty nonviruliferous insects were microinjected with purified GFP or P7-1 proteins. Insect GAPDH was served as the reference protein. The relative intensities of bands of different proteins were determined using ImageJ. Data represent three biological replicates. (M) Immunoprecipitation analysis of the ubiquitination of MyD88 in Sf9 cells. Cells were transfected with MyD88-Flag, Ub-HA, together with SfREL-His or P7-1 as indicated.

To further elucidate the activation and function of SfREL during viral infection, Y2H and GST pull-down assays were conducted. The results demonstrated that both MyD88 and SfREL interacted with viral nonstructural protein P7-1 (Fig 5A–5C). Y2H assays further revealed that P7-1 interacted with the RING domain-containing N-terminal fragment, but not with the C-terminal fragment of SfREL (Fig 5D and 5E). Subsequently, the baculovirus expression system in Sf9 cells was used to investigate the relationship among P7-1, SfREL, and MyD88. When expressed individually, MyD88-Flag was associated with the cellular membrane, P7-1 formed fibrillar structures in the cytoplasm, while SfREL-His was distributed in the nucleus of Sf9 cells (Fig 5F). However, co-expression of SfREL-His and MyD88-Flag led to the redistribution of SfREL-His from the nucleus into the cytomembrane (Fig 5G). P7-1 and SfREL-His were colocalized in the fibrillar structures (Fig 5H), whereas P7-1 and MyD88-Flag were colocalized in the cytomembrane in the co-expressed cells (Fig 5I). The triple expression of P7-1, SfREL-His, and MyD88-Flag resulted in their co-localization in the cytomembrane (Fig 5J). These results suggest P7-1, MyD88, and SfREL potentially form the complex during viral infection in insect vectors.

An *in vitro* ubiquitination assay was performed to determine whether P7-1 is a substrate of SfREL. Interestingly, P7-1 fused with GST tag was not ubiquitinated by SfREL when E1, E2, and ubiquitin were present (Fig 5K). We then investigated whether P7-1 alone could activate SfREL expression. Microinjection of third-instar nymphs of *S*. *furcifera* with purified P7-1 proteins resulted in the increased accumulation of SfREL compared to the GFP control (Fig 5L). We then examined whether P7-1 could promote SfREL-mediated ubiquitinated degradation of MyD88 in Sf9 cells. Upon treatment with 50 μM MG132, a higher accumulation of ubiquitinated MyD88-Flag was observed in Sf9 cells co-expressing SfREL, MyD88-Flag, and P7-1 compared to cells co-expressing SfREL and MyD88-Flag, or cells co-expressing P7-1 and MyD88-Flag (Fig 5M). These findings collectively demonstrate that SRBSDV P7-1 promotes SfREL-mediated ubiquitinated degradation of MyD88, thus attenuating the Toll antiviral pathway in insect vectors.

### The recruitment of SfREL into tubular or fibrillar structures of P7-1 in insect vector facilitates viral propagation

**Fig 6:**
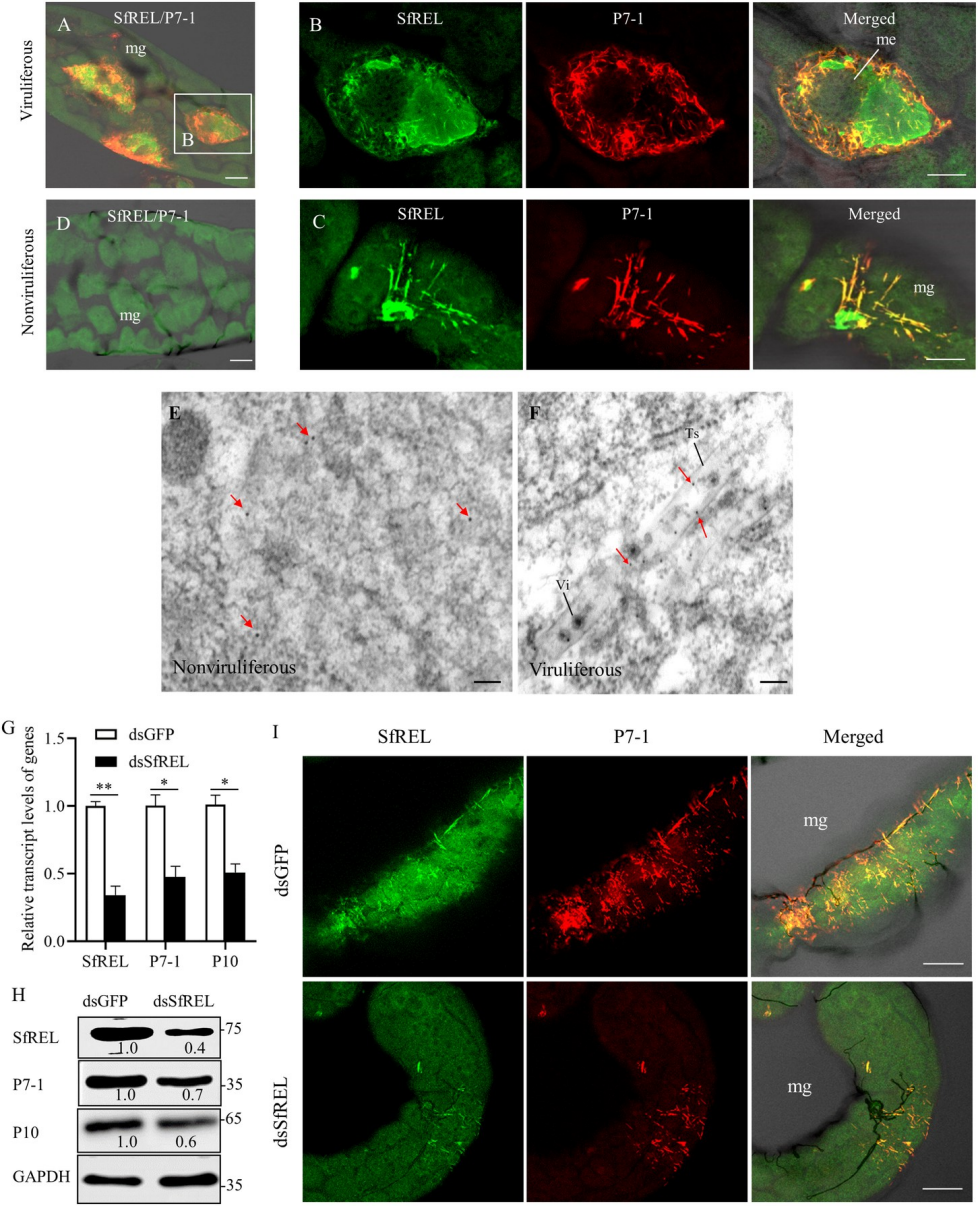
SfREL colocalizes with SRBSDV P7-1 to benefit viral infection in *S*. *furcifera*. (A-D) The colocalization of SfREL with SRBSDV P7-1 in virus-infected midgut, as determined by immunofluorescence microscopy. The intestines of viruliferous (A-C) and nonviruliferous (D) insects were immunolabeled with SfREL-FITC (green) and P7-1-rhodamine (red). mg, midgut; me, midgut epithelium. Bars, 5 μm. (E and F) Immunogold labeling of SfREL in P7-1-formed structures in virus-infected midgut. The intestines of nonviruliferous (E) and viruliferous (F) insects were immunolabeled with SfREL antibody as the primary antibody, followed by treatment with 15-nm gold particle-conjugated IgG as the secondary antibody. Red arrows indicate gold particles. Ts, tubular structure; Vi, virions. Bars, 100 nm. (G and H) The relative transcript (G) and accumulation (H) levels of SfREL, P7-1, and P10 in 30 dsGFP- or dsSfREL-treated viruliferous insects, as determined by RT-qPCR and western blot assays. Means (± SD) are shown from three biological replicates. *, *P*<0.05; **, *P*<0.01. Insect GAPDH was served as reference protein. The relative intensities of bands of different proteins were determined using ImageJ. (I) Effect of knocking down *SfREL* expression on the colocalization of SfREL and P7-1 in the intestines. The intestines of dsSfREL- and dsGFP-treated viruliferous insects were immunolabeled with SfREL-FITC (green) and P7-1-rhodamine (red), mg, midgut. Bars, 5 μm.

SRBSDV takes advantage of virus-associated tubular or fibrillar structures composed of the nonstructural membrane protein P7-1 to spread throughout the body of *S*. *furcifera* [28]. We further investigated how SfREL was involved in the formation of these tubular or fibrillar structures during viral infection in insect vectors. At 6 days padp, immunofluorescence microscopy of virus-infected midguts of *S*. *furcifera* showed the extensive colocalization of SfREL with fibrillar structures of P7-1 (Fig 6A–6C). However, only low staining was observed in nonviruliferous controls (Fig 6D). Immunoelectron microscopy further confirmed that SfREL antibody specifically reacted with virus-associated tubular or fibrillar structures in the virus-infected midgut of *S*. *furcifera* (Fig 6E and 6F). RT-qPCR and western blot assays revealed a significant decrease in the accumulation levels of viral proteins in dsSfREL-treated insects (Fig 6G and 6H). Immunofluorescence microscopy revealed that P7-1 was restricted to limited midgut regions in dsSfREL-treated insects (Fig 6I). These findings collectively suggest that viral infection activates SfREL to support the assembly of tubular or fibrillar structures of P7-1, facilitating viral propagation in insect vectors.

### Defensin possesses antiviral activity by directly interacting with viral major outer capsid protein P10

**Fig 7:**
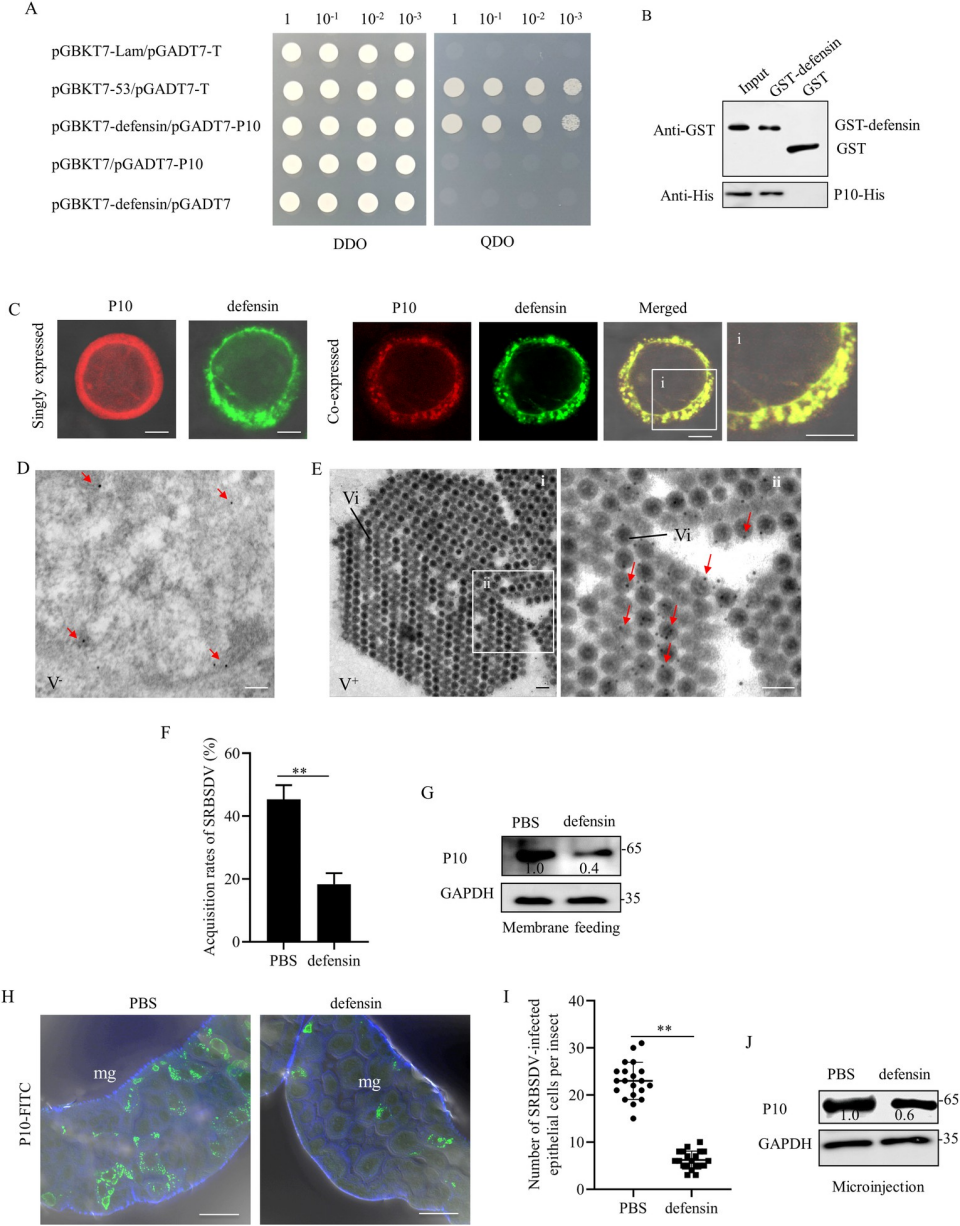
Defensin exhibits antiviral activity by interacting with SRBSDV P10. (A) Interaction between defensin and P10 in Y2H assays. Transformants were plated on either DDO or QDO. DDO, SD/-Trp-Leu medium. QDO, SD/-Trp-Leu-His-Ade medium. (B) Interaction between defensin and P10 in GST pull-down assay. GST-defensin was incubated with glutathione-Sepharose beads. P10-His was then added to the beads, followed by western blot assay to detect P10-His bound to GST-defensin. (C) Defensin-His and P10 singly expressed or co-expressed in Sf9 cells. Cells were respectively immunolabeled with His-Alexa Fluor 488 (green) or P10-Alexa Fluor 555 (red). The images were merged under a background of transmitted light. Panel i is the enlarged image of the boxed areas in left panel. Bars, 5 μm. (D and E) Immunoelectron microscopy showing the association of defensin with SRBSDV particles in virus-infected midgut. The intestines of nonviruliferous (D) and viruliferous (E) insects were immunolabeled with defensin antibody as the primary antibody, followed by treatment with 15-nm gold particle-conjugated IgG as the secondary antibody. Panel E-ii was the enlarged image of the boxed area in panel E-i. Red arrows indicate gold particles. V^-^, nonviruliferous; V^+^, viruliferous; Vi, virions. Bars, 100 nm. (F) Effects of membrane feeding of the mixture of purified defensin proteins and viral particles on the acquisition rates of SRBSDV in *S*. *furcifera*. The PBS buffer mixed with purified SRBSDV particles served as the control. Data are presented as means (± SD) of three independent biological replicates and each replicate contains 30 insects. **, *P*<0.01. **(**G) Effects of defensin treatment through membrane feeding on the accumulation of SRBSDV P10 in 30 insects, as determined by western blot assay. (H) The intestines of insects microinjected with purified defensin or PBS mixed with purified SRBSDV particles were immunolabeled with P10-FITC (green). mg, midgut. Bars, 5 μm. (I) The average number of epithelial cells infected with SRBSDV in insects microinjected with defensin or PBS mixed with purified SRBSDV particles. Bars represent means ± SD from more than 20 individual cells. **, *P*<0.01. (J) Effects of the microinjected defensin on the accumulation of SRBSDV P10 in 30 insects, as determined by western blot assay. Insect GAPDH in G and J served as the reference of total proteins. The relative intensities of bands of P10 protein were determined using ImageJ.

We then examined how Toll signaling pathway downstream factor defensin possessed antiviral activity. Y2H and GST pull-down assays showed the interaction of defensin with P10 of SRBSDV (Fig 7A and 7B). Subsequently, the baculovirus expression system in Sf9 cells was used to investigate the relationship between P10 and defensin. When expressed individually, defensin formed small punctate inclusions in the cytoplasm, while P10 was diffusely distributed in the cytoplasm of Sf9 cells (Fig 7C). Co-expression of defensin and P10 led to the recruitment of P10 to the punctate inclusions of defensin in the cytoplasm (Fig 7C). Immunoelectron microscopy further confirmed that defensin antibody specifically reacted with viral particles in the virus-infected midgut epithelium of *S*. *furcifera* (Fig 7D and 7E). Together, defensin could directly bind to viral particles in *S*. *furcifera*.

To further investigate how defensin possessed antiviral activity, the purified defensin proteins were mixed with purified SRBSDV particles, and then were delivered into *S*. *furcifera* by membrane feeding. After membrane feeding for 6 days, RT-PCR assay showed that defensin treatment significantly reduced the acquisition rate of SRBSDV in *S*. *furcifera* (Fig 7F). Western blot assay confirmed that the fed defensin proteins led to the reduced accumulation of P10 of SRBSDV in *S*. *furcifera* (Fig 7G), suggesting that defensin effectively inhibits the acquisition of viral particles into vector midgut epithelium for propagation. Alternatively, the mixture of purified defensin proteins and viral particles was microinjected into the bodies of *S*. *furcifera*.

After microinjection for 4 days, immunofluorescence microscopy showed that defensin significantly inhibited the infection of SRBSDV in insect midgut (Fig 7H and 7I). Western blot assay confirmed that the microinjected defensin proteins led to the reduced accumulation level of P10 in *S*. *furcifera* (Fig 7J), confirming the vital role of defensin in combating SRBSDV infection via directing binding to viral particles in insect vectors.

## Discussion

**Fig 8:**
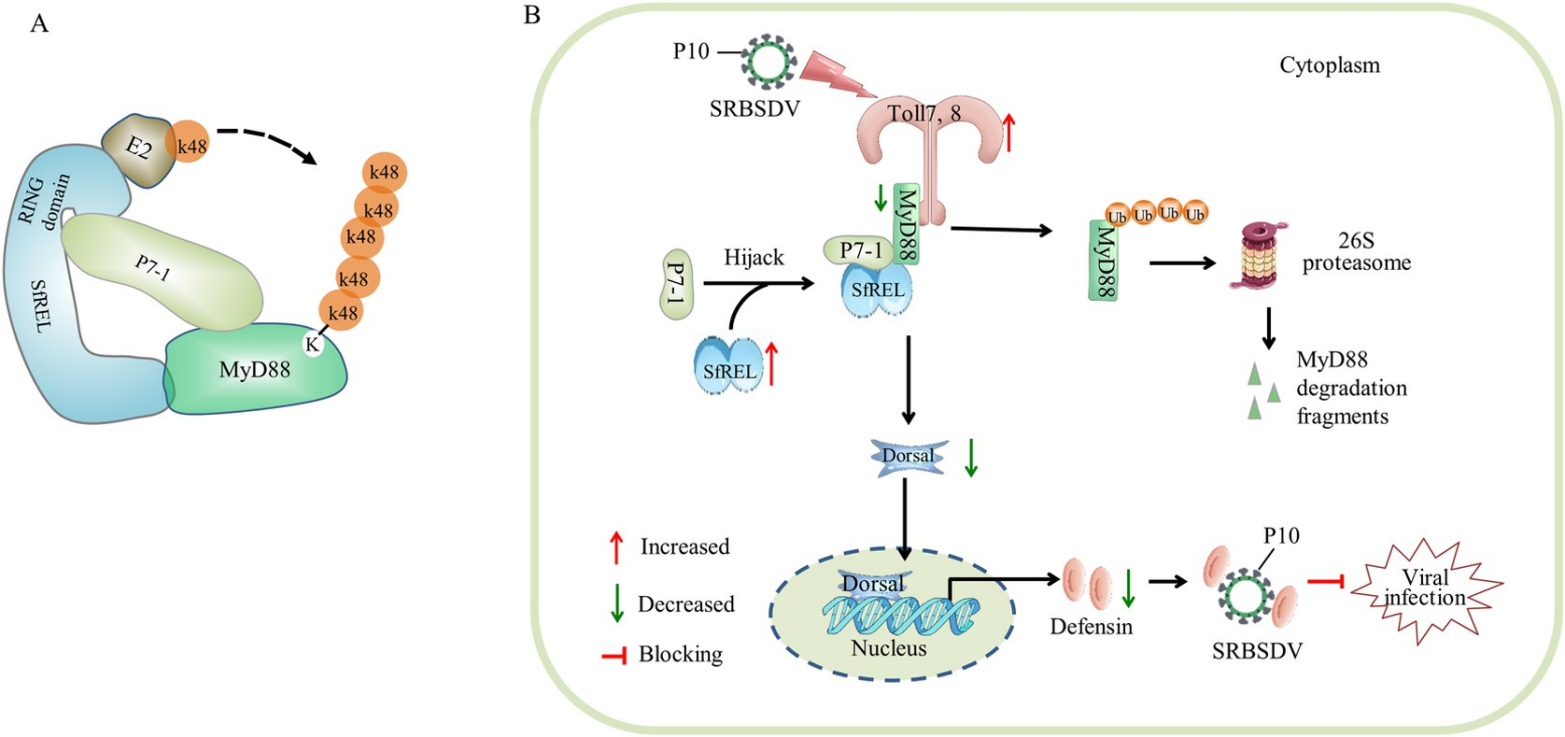
Proposed model of viral proteins attenuating antiviral Toll pathway via promoting RING E3-mediated ubiquitinated degradation of MyD88. (A) Interaction model of SfREL, E2, MyD88, P7-1 and Ub-K48. (B) Upon SRBSDV infection in planthopper vectors, the recognition of viral PAMPs activates the upstream Toll7 and Toll8 receptors. Subsequently, viral infection suppresses the downstream MyD88-Dorsal-defensin cascade, thereby attenuating the Toll antiviral immune response. Toll pathway-induced defensin directly interacts with viral major outer capsid protein P10 and thus blocks effective viral infection in planthopper vector. Meanwhile, SRBSDV P7-1 activates and promotes RING E3-meidated ubiquitinated degradation of MyD88 in a proteasome-dependent manner, finally attenuating antiviral Toll pathway to promote the fitness of the viruliferous insect vectors.

The long-term association between arboviruses and their insect vectors involves evolutionary trade-offs that maintain a balance between insect fitness cost and persistent viral transmission. During persistent viral infection in insect vectors, arboviruses induce immune homeostasis to modulate vector-virus coexistence [38]. However, the mechanisms by which arboviruses modulate immune homeostasis remain poorly understood. In recent years, the planthopper-transmitted SRBSDV has caused widespread outbreaks and significant yield losses in Asian rice-growing countries [39]. In this study, we show that defensin directly interacts with viral major outer capsid protein P10 and thus binds to viral particles, finally blocking effective viral infection in planthopper *S*. *furcifera* (Fig 8). We further reveal that SRBSDV infection in *S*. *furcifera* activates the upstream Toll7 and Toll8 expression but suppresses the downstream MyD88-Dorsal-defensin cascade, resulting in the attenuation of the Toll antiviral immune response (Fig 8). Importantly, the attenuation of the Toll antiviral immune response facilitates viral transmission without causing noticeable insect fitness cost. We demonstrate that knockdown of *MyD88* expression further suppresses the Toll antiviral immune response but significantly promotes the propagation of SRBSDV beyond the pathogenic threshold in viruliferous *S*. *furcifera*, ultimately leading to a detectable insect fitness cost. Our findings show that an appropriate Toll antiviral response mediates a metastable balance between viral accumulation and pathogenicity in insect vectors, enabling viral persistence and efficient spread in nature. Thus, both the insect and the virus reach a metastable equilibrium that defines the state of persistent infection. These findings have broad implications for understanding the establishment of persistent infection of plant viruses in insect vectors in nature.

Numerous viruses activate the expression of genes related to Toll signaling pathway to mediate antiviral effects in mosquitoes and *Drosophila* [13,23,37]. However, the molecular mechanisms by which viruses antagonize the Toll antiviral response in insects are still poorly understood [13,23,37]. In invertebrates, the Toll pathway primarily relies on pattern recognition receptors (PRRs), known as Toll receptors, which recognize pathogen-associated molecular patterns (PAMPs) to induce downstream effectors against viral infection [23,40]. Consequently, the recognition of viral PAMPs by Toll PRRs activates the upstream Toll receptors [41,42]. Once the pathway is activated, the subsequent intracellular MyD88-Tube-Pelle-Cactus-Dorsal-AMPs signaling cascade is initiated [43–47]. In general, the adaptor protein MyD88 recruits Tube and Pelle to form a complex, which dissociates Cactus from the complex of Cactus and Dorsal, thereby initiating the translocation of the transcription factor Dorsal into the nucleus for regulating the expression of AMPs [14]. Recent report shows that RSV can antagonize the Toll antiviral response in planthopper vectors through competitively binding to Dorsal [24]. Toll pathways in insects exert strong evolutionary pressure on viruses, and viral evasion of the Toll immune response may occur in a conserved manner [13,23,37]. MyD88, a vital conserved adaptor protein, is considered as a central player in regulating the Toll signaling pathway [19,48,49]. In our study, we discover that the nonstructural protein P7-1 of SRBSDV effectively promotes the E3 ubiquitin ligase SfREL-mediated ubiquitinated degradation of the adaptor protein MyD88 in a proteasome-dependent manner (Fig 8). We deduce that virus-mediated the reduced MyD88 accumulation leads to the suppression of MyD88-Tube-Pelle complex formation, which finally inhibits the translocation of Dorsal into the nucleus for regulating the expression of *defensin* (Fig 8). Targeting MyD88 for pathogen antagonism of the Toll immune response pathway may represent a universal mechanism for ensuring the persistent transmission of arboviruses and other pathogens by their respective insect vectors.

The RING domain of E3 ubiquitin ligase is responsible for facilitating the transfer of ubiquitin from an E2 ubiquitin-conjugating enzyme to the target protein [37]. Our results confirm that an E3 ligase inactive mutant, in which His-61 is replaced by Tyr in the C3HC4-type RING domain, abolishes the E3 ubiquitin ligase activity of SfREL in MyD88 ubiquitination. Typically, SfREL facilitates the attachment of K48-linked polyubiquitin chains to target proteins for subsequent proteasomal degradation. Interestingly, the interaction among P7-1, SfREL, and MyD88 promotes SfREL-mediated ubiquitinated degradation of MyD88 through K48-linked polyubiquitination. P7-1 interacts with the RING domain-containing N-terminus of SfREL and MyD88, while MyD88 interacts with the C-terminus of SfREL. Thus, P7-1 acts as a bridge between SfREL and MyD88 but does not affect SfREL-mediated MyD88 ubiquitination (Fig 7A). Furthermore, SfREL also is involved in the formation of tubular structures of P7-1, facilitating efficient spread of SRBSDV within insect vectors [25–30]. Therefore, SfREL could coordinate the assembly of P7-1-formed structures and the ubiquitinated degradation of MyD88 in insect vectors, thus acting as a pro-viral factor during persistent viral transmission by insect vectors.

Recently, we have demonstrated that SRBSDV can activate the pro-viral mitophagy pathway, preventing extensive apoptotic response and reducing apoptosis-induced fitness costs in insect vectors [32]. Furthermore, the virus-induced siRNA antiviral pathway is a universal mechanism for controlling viral infection in insect vectors [9,27]. It would be valuable to investigate whether there is crosstalk between these immune response systems and how viruses modulate the homeostasis of these immune responses during persistent viral infection in insect vectors. In *Drosophila*, the Toll7 receptor of the Toll pathway activates antiviral autophagy against vesicular stomatitis virus and rift valley fever virus through pathogen recognition [50,51]. In our study, we have discovered that SRBSDV P7-1 can trigger pro-viral mitophagy in insect vectors [32]. Consequently, P7-1 can synergistically regulate mitophagy and the Toll pathway, achieving a balance between these two immune responses. To the best of our knowledge, this is the first evidence showing that a viral protein can simultaneously activate pro-viral mitophagy and attenuate the antiviral Toll pathway to maintain persistent viral infection in insect vectors.

## Materials and methods

### Ethics statement

The antibodies SfREL, MyD88, Dorsal, and defensin were produced by Genscript USA Innovation Company (Nanjing), which is approved by the Science Technology Department of Jiangsu Province, China with approval number SYXK (Su) 2018–0015.

### Insects, viruses and antibodies

Nonviruliferous *S*. *furcifera* adults were collected from rice fields in Nanping city, Fujian Province, China, and propagated on TN-1 rice seedlings in cages at 25 ± 1°C with 75 ± 5% relative humidity and a 16-hour light/8-hour dark cycle. SRBSDV-infected rice plants were initially collected from Nanping city and propagated through transmission by *S*. *furcifera*. Sf9 cells were cultured and maintained in Sf900 III growth medium (Gbico, 12658019).

Rabbit polyclonal antibodies against SfREL, MyD88, Dorsal, and defensin of *S*. *furcifera* were prepared by GenScript Biotech Corporation in Nanjing, China. The process was approved by the Science Technology Department of Jiangsu Province, China. Polyclonal antibodies against P7-1 and P10 of SRBSDV were prepared as previously described [28]. These polyclonal antibodies were directly conjugated to fluorescein isothiocyanate (FITC), rhodamine, or Alexa Fluor 647 (Thermo Fisher Scientific) according to the manufacturer’s instructions. Antibodies against Flag-Alexa Fluor 488 or 555, and His-Alexa Fluor 488 or 555 were purchased from Thermo Fisher Scientific (MA1-142-A488, MA1-142-A555, and MA1-21315-A555) and Abcam (ab237336). The mouse monoclonal antibodies against GST (HT601) and histone H3 (HL102-01) were purchased from Transgene Biotech, and the mouse monoclonal antibodies against the 6×His tag (D110002), HA (D110004), and Flag (D191041) were purchased from Sangon Biotech.

### Y2H assay

The Y2H screening experiment was conducted using the Matchmaker Gal4 Two-Hybrid System3 (Clontech) according to the manufacturer’s instructions. The open reading frame (ORF) of *MyD88* from *S*. *furcifera* was amplified and cloned into the bait plasmid pGBKT7 to screen potential interacting proteins from the cDNA library of *S*. *furcifera*, which was constructed using the prey plasmid pGADT7 [30]. Positive clones were selected in SD quadruple dropout (QDO) medium (SD/-Ade/-His/-Leu/-Trp) after library screening. Prey plasmids were then isolated for sequencing.

To investigate the interactions among MyD88, SfREL, and P7-1, and the interactions between defensin and P10, the ORFs of *P7-1*, *P10*, *MyD88*, *SfREL*, and *defensin*, as well as the N-terminal segment (amino acids 1–200, SfREL-N) and C-terminal segment (amino acids 201–661, SfREL-C) of SfREL, were individually amplified and cloned into the bait plasmid pGBKT7 or the prey plasmid pGADT7. The primers used for amplification are listed in S1 Table. The bait and prey plasmids were co-transformed into the yeast strain AH109. The β-galactosidase activity was assessed in QDO/X-α-gal culture medium. The positive control pGBKT7-53/pGADT7-T and negative control pGBKT7-Lam/pGADT7-T were also transformed in the same manner.

### Y1H assay

To identify whether Dorsal interacted with the promoter of *defensin*, the 2,000 bp putative promoter sequences of *defensin* of *S*. *furcifera* were downloaded from genomic data (GCA_017141385.1) from the NCBI website. The putative Dorsal-binding motifs of *defensin* were predicted using the JASPAR database (http://jaspardev.genereg.net/)), and subsequently cloned into the pHIS2 vector as the reporter vector. The ORF of *Dorsal* was inserted into the pGADT7 vector to construct the prey vector (pGADT7-Dorsal). The reporter vector was co-transformed into the yeast strain Y187 with pGADT7-Dorsal and plated on DDO (SD/-Leu/-Trp) and TDO (SD/-Leu/-Trp/-His) media containing 200 mM 3-AT for the yeast one-hybrid assay. Empty vector pHIS2 and pGADT7-Dorsal were also transformed as negative controls. Images were taken after 4 days of incubation at 30°C.

### EMSA

The ORF of *Dorsal* was cloned into the pEASY-Blunt E1 plasmid to create Dorsal-His fusion protein. The expressed Dorsal-His was then purified using nickel-nitrilotriacetic acid (Ni-NTA) resin (Qiagen). The *defensin*-Cy5-probe containing the putative Dorsal-binding motif sequences (ACTGGAATTACCCGGGAAATGAGATTTTCCAG) was designed. The mutant probe containing the sequences (GGAGAGTCCTCAG) also was designed. All the probes were synthesized and labeled with Cy5 (Sangon Biotech). For the EMSA, the probe (20 fmol), purified Dorsal-His proteins (100 μg), and Poly(dI-dC) (10 μg) (Thermo Fisher Scientific) were incubated at 4°C for 30 min in EMSA buffer (10 mM Tris-HCl pH7.5, 0.25 mM DTT, 5 mM MgCl2, 10 mM KCl). The reaction mixtures were then resolved on 5% non-denaturing polyacrylamide gels. Finally, the Cy5-labeled DNA on the gel was detected using an infrared spectrum imaging system (LiCOR Odyssey).

### GST pull-down assay

The ORFs of *MyD88*, *defensin* and *SfREL* were individually cloned into the pGEX-4T-1 plasmid to fuse with the GST tag. The ORFs of *P7-1*, *P10* and *SfREL* were individually cloned into the pEASY-Blunt E1 plasmid to fuse with the 6×His tag. The primers used for cloning are listed in S1 Table. GST-SfREL, GST-defensin or GST-MyD88 were bound to GST-Sepharose 4B beads (GE Healthcare, 17-0756-01) at 4°C for 3 h, followed by centrifugation at 100 × g for 5 min to remove the supernatant. His-P7-1, His-P10 or SfREL-His was added to the beads, and the mixture was incubated at 4°C for 2 h. The mixture was then centrifuged at 100 × g for 5 min and washed with wash buffer (300 mM NaCl, 10 mM Na2HPO_3_, 2.7 mM KCl, 1.7 M KH_2_PO_4_). The immunoprecipitated proteins were detected by western blot assay using a GST-tagged antibody and a His-tagged antibody, respectively.

### Detection of viral or insect gene expression by RT-qPCR and western blot assays

The effects of SRBSDV infection on the expression of genes associated with the Toll pathway or RING E3 in *S*. *furcifera* were examined by RT-qPCR assays. Approximately 500 third instar nymphs of *S*. *furcifera* were allowed to feed on SRBSDV-infected rice plants for 2 days and then transferred to healthy rice seedlings. At 6 days padp, total RNAs were isolated from 30 virus-free or virus-infected *S*. *furcifera* individuals using TRIzol reagent (Thermo Fisher Scientific). RT-qPCR assays were performed using the SYBR Green PCR Master Mix Kit (GenStar) in the QuantStudio 5 Real-Time PCR System (Applied Biosystems). The relative transcript levels of genes related to the Toll pathway or RING E3 were calculated using the 2^-ΔΔCT^ method [52]. The eukaryotic translation elongation factor 1 (EF1) of *S*. *furcifera* was used as the internal reference for normalizing gene expression levels. The primers used in the RT-qPCR assays are listed in S1 Table. The assays were performed with three biological replicates.

The effects of SRBSDV infection on the accumulation of SRBSDV proteins, Toll pathway-related proteins, or RING E3 in *S*. *furcifera* were also examined by western blot assays. Third instar nymphs of *S*. *furcifera* were allowed to feed on SRBSDV-infected rice plants for 2 days and then transferred to healthy rice seedlings. At 6 days padp, total proteins were extracted from 30 virus-free or virus-infected *S*. *furcifera* individuals. Moreover, the nuclear proteins were extracted from 30 virus-free or virus-infected *S*. *furcifera* individuals using a Nuclear and Cytoplasmic Extraction kit (Beyotime). Antibodies against SRBSDV P7-1, SRBSDV P10, SfREL, MyD88, Dorsal, and defensin were used as primary antibodies, and goat anti-rabbit IgG-peroxidase (Sigma-Aldrich) was used as the secondary antibody. The accumulation level of GAPDH served as the reference of total proteins using GAPDH-specific antibody (Sigma-Aldrich). The histone H3 served as the reference of nuclear proteins using H3-specific antibody (Transgene Biotech). The proteins were visualized using the Luminata Classico Western HRP Substrate (Millipore) and imaged using the Molecular Imager ChemiDoc XRS+ System (Bio-Rad). ImageJ software (https://imagej.nih.gov/ij/) was used to measure the band intensities of the proteins.

### Immunofluorescence microscopy

To visualize the association of SfREL with P7-1 of SRBSDV in the midgut, the intestines of 30 nonviruliferous and viruliferous insects, or 30 dsSfREL- or dsGFP-treated viruliferous insects, were dissected. The samples were fixed in 4% paraformaldehyde in PBS (v/v) for 2 h and permeabilized in 0.2% Triton-X (v/v) for 1 h. Then, the samples were immunolabeled with P7-1 antibody directly conjugated to rhodamine (P7-1-rhodamine) and SfREL antibody directly conjugated to FITC (SfREL-FITC) at a concentration of 0.5 μg/μL. The immunostained tissues were analyzed using a Leica TCS SPE inverted confocal microscope.

### Immunoelectron microscopy

To visualize the distribution of SfREL or defensin during SRBSDV infection in the midgut of *S*. *furcifera*, the intestines from both nonviruliferous and viruliferous *S*. *furcifera* insects were fixed with 2% glutaraldehyde and 2% paraformaldehyde in PBS for 2 h at room temperature. Following fixation, the samples were dehydrated using a graded ethanol series at -20°C and embedded in LR gold resin (Bioscience). Polymerization was allowed to proceed for 72 h at -20°C. The samples were then sectioned, and the ultrathin sections were immunolabeled with SfREL or defensin antibody (0.5 μg/μL) as the primary antibody. Subsequently, the sections were treated with goat anti-rabbit IgG conjugated with 15-nm diameter gold particles (0.5 μg/μL; Sigma-Aldrich) as the secondary antibody. Finally, the ultrathin sections were analyzed using a transmission electron microscope (H-7650; Hitachi).

### Silencing genes related to Toll pathway or RING E3 in *S*. *furcifera*

The dsRNAs targeting sequences of approximately 500 bp or full lengths of *Toll7* (dsToll7), *Toll8* (dsToll8), *Dorsal* (dsDorsal), *defensin* (dsdefensin), *MyD88* (dsMyD88), *SfREL* (dsSfREL), and *GFP* (dsGFP) were synthesized *in vitro* using the T7 RiboMAX Express RNAi System from Promega Biotech. The synthesis was carried out following the manufacturer’s protocol. Approximate 600 third-instar nymphs of *S*. *furcifera* were allowed to feed on infected rice plants for 2 days. Afterward, 0.5 μg/μL dsRNAs were microinjected into both nonviruliferous and viruliferous insects, which were then transferred to healthy rice seedlings for recovery. At 6 days padp, the efficiency of gene silencing was assessed through RT-qPCR and western blot assays. For total RNA extraction, a random mixture of thirty insects from each dsRNA treatment was used. The dsGFP treatment served as the control. Subsequently, RT-qPCR assays were conducted to evaluate the replication of viral RNAs of *P10* and *P7-1*. Meanwhile, 30 insects from each dsRNA treatment were randomly mixed for total protein extraction. The antibodies against MyD88, defensin, Dorsal, SfREL, P7-1, and P10 were used in western blot assays to assess the Toll pathway and viral propagation. Insect GAPDH was utilized as the reference protein. For signal quantification, the ImageJ software (https://imagej.nih.gov/ij/) was used. Each replicate was performed using a pool of 30 dsRNA-treated insects, and the experiment included three replicates for both RT-qPCR and western blot assays. S1 Table shows the primers used in the RT-qPCR assays.

To evaluate insect fitness, approximate 200 third instar nymphs of *S*. *furcifera* were allowed to feed on SRBSDV infected or healthy rice plants for 2 days. Then, they were microinjected with either 0.5 μg/μl dsGFP or dsMyD88. Simultaneously, approximately 200 third instar nymphs of *S*. *furcifera* were microinjected with 0.5 μg/μl dsGFP or dsMyD88, which served as the control. The survival of the insects were recorded daily, and each treatment was conducted with three biological replicates.

To investigate viral acquisition rates, 30 third instar nymphs microinjected with dsMyD88- and dsGFP were placed on SRBSDV-infected plant for two days, then transferred to healthy rice seedlings. At 6 days padp, the acquisition rates of SRBSDV by *S*. *furcifera* were assessed using RT-qPCR assays. Three replicates were conducted and each replicate contains 30 insects.

To investigate viral transmission rates, 30 dsMyD88- and dsGFP-treated viruliferous *S*. *furcifera* adults were placed in glass tubes containing a single rice seedling. The insects were maintained for 10 days, with the seedlings replaced daily, as described previously [53]. Three replicates were conducted and each replicate contains 30 insects. The insects were collected and analyzed by RT-PCR assay at 16 days padp. The plants inoculated with the confirmed viruliferous *S*. *furcifera* were subjected to RT-PCR detection 15 days later. The transmission rates of SRBSDV by *S*. *furcifera* were calculated as the percentage of RT-PCR positive plants out of the total number of plants.

### Microinjection of purified protein into *S*. *furcifera*

The ORFs of *defensin*, *P7-1*, and *GFP* were cloned into pEASY-Blunt E1 (TransGen Biotech, CE111-01) for fusion with a His tag. The recombinant proteins were expressed in *Escherichia coli* strain Rosetta and purified following the methods as previously described [54]. Approximately 200 third instar nymphs of *S*. *furcifera* were allowed to feed on infected rice plants for 2 days. Viruliferous *S*. *furcifera* individuals were then microinjected with purified defensin or GFP proteins at a concentration of 300 μg/mL. At 4 days post-microinjection, the insects were tested for the relative transcript levels of *P10* using RT-qPCR assays and the accumulation of P10 using western blot assays, as described earlier. To investigate the role of viral proteins in the activation of SfREL accumulation, nonviruliferous third instar *S*. *furcifera* individuals were microinjected with purified P7-1 or GFP proteins at a concentration of 300 μg/mL. At 4 days post-microinjection, the insects were tested for the relative transcript levels of *SfREL* using RT-qPCR assays and the accumulation of SfREL using western blot assays.

To investigate the effect of defensin on the infection of SRBSDV, purified defensin proteins at a concentration of 300 μg/mL were mixed with purified SRBSDV particles at a concentration of 10 μg/mL, and then were microinjected into nonviruliferous third instar *S*. *furcifera* individuals using a Nanoject II Auto-Nanoliter Injector (Spring). The nonviruliferous third instar *S*. *furcifera* individuals microinjected with PBS mixed with purified SRBSDV particles served as the control. At 4 days post-microinjection, the insects were dissected and immunolabeled with P10 antibody conjugated to FITC (P10-FITC), and then examined by immunofluorescence microscopy. Meanwhile, the accumulation levels of P10 in insects microinjected with purified defensin proteins or PBS were determined by western blot assay.

To further investigate the effect of defensin on the infection of SRBSDV, a membrane-feeding method was employed to deliver a mixture of purified defensin proteins and viral particles to *S*. *furcifera* [55]. Third-instar nymphs of *S*. *furcifera* were allowed to feed with the mixture of purified defensin proteins and purified viral particles that held between two layers of stretched parafilm covering one open end of the chamber for 1–2 days. Subsequently, the insects were transferred on healthy rice seedlings. The PBS buffer mixed with purified SRBSDV particles served as the control. After membrane feeding for 6 days, the insects were tested for the presence of SRBSDV using RT-PCR assays. Meanwhile, the accumulation levels of P10 of SRBSDV in insects were assessed via western blot assay.

### Baculovirus expression assays

The ORFs of *P7-1*, *P10*, *MyD88*, *defensin* and *SfREL* were amplified using reverse primers that contained the coding sequences for the His-tag and Flag-tag. The PCR products of P7-1, P10, SfREL-His, defensin-His, and MyD88-Flag were purified and cloned into the pDEST8 vector (Thermo Fisher Scientific) to generate recombinant baculoviruses. Recombinant bacmids were produced by transforming *E*. *coli* DH10Bac (Thermo Fisher Scientific) with the recombinant baculoviruses. The purified recombinant bacmids were then transfected into Sf9 cells using Cellfectin II (Thermo Fisher Scientific). At 48 h post-infection (hpi), the cells were fixed in 4% paraformaldehyde in PBS for 30 minutes and permeabilized in 0.2% Triton X-100 (v/v) for 15 minutes. Subsequently, the cells were immunolabeled with P7-1 antibody directly conjugated to FITC, rhodamine, or Alexa Fluor 647 (designated as P7-1-FITC, P7-1-rhodamine, or P7-1-Alexa Fluor 647) or P10 antibody directly conjugated to rhodamine (P10-rhodamine). Additionally, Flag antibody conjugated to Alexa Fluor 488 or 555 and His antibody conjugated to Alexa Fluor 488 or 555 were used. The cells were then processed for immunofluorescence microscopy.

### Ubiquitination assays

The ubiquitination assays *in vitro* were performed according to the protocol of the E3 Ligase Auto Ubiquitination Assay Kit (Abcam). To investigate whether SfREL had ubiquitin E3 ligase activity, E1-His, E2-His, GST-SfREL, and GST-SfREL (H61Y) were expressed in *E*. *coli* BL21 strain and then purified. GST-SfREL or GST-SfREL (H61Y) was subsequently incubated with E1-His, E2-His, ubiquitin, DTT (50 mM), inorganic pyrophophatase solution (IPP) (100 U/mL in 20 mM Tris-Cl, pH 7.5), and 10 × E3 ligase buffer (50 mM Tris–HCl pH 7.4, 2 mM ATP, 5 mM MgCl_2_) at 37°C for 1 h. Pre-incubated GST protein served as the negative control. Reactions were stopped by adding 5 × protein loading sample buffer followed by boiling for 10 min. Antibodies against ubiquitin (Abcam, ab134953) and GST were then employed to detect the ubiquitinated proteins in western blot assays. To investigate SfREL-mediated MyD88 or P7-1 ubiquitination, GST-MyD88, GST-P7-1, GST-SfREL, GST-SfREL (H61Y), SfREL-HA, and SfREL (H61Y)-HA were expressed in *E*. *coli* BL21 strain and then purified. GST-MyD88 or GST-P7-1 was subsequently incubated with E1-His, E2-His, SfREL-HA, ubiquitin, or K48-ubiquitin (a variant ubiquitin with a Lys-to-Arg change at position 48), DTT, IPP, and 10 × E3 ligase buffer at 37°C for 1 h as described above. Pre-incubated GST protein was used as the negative control. Reactions were stopped by adding 5 × protein loading sample buffer followed by boiling for 10 min. Antibodies against ubiquitin (Abcam, ab134953), ubiquitin (K48) (Abmart, T55964S), His-Tag (Sangon Biotech, D110002), GST-Tag (Sangon Biotech, D110271), and HA-Tag (Sangon Biotech, D110004) were then utilized to detect the ubiquitinated proteins and input proteins using western blot assays.

We then tested whether SfREL or P7-1 was involved in the degradation of MyD88 *in vivo*. Sf9 cells were first co-transfected with recombinant bacmids expressing GFP or SfREL-His and ubiquitin-HA or MyD88-Flag. Alternatively, the Sf9 cells were treated with 5 μM MG132 for 4 h [56]. Then cells were harvested using NP-40 lysis buffer and incubated with Flag beads (AlpalifeBio, KTSM1338) for 3 h at 4°C. All beads were collected via centrifugation for 1 min at 12, 000 × g. The pellets were washed 4–5 times, and then 5× loading buffer was added to the samples. Western blot assay was performed to analyze the immunoprecipitants. The input lysates from whole cells served as the control. To examine whether P7-1 promoted SfREL-mediated degradation of MyD88, Sf9 cells were co-transfected with recombinant bacmids expressing P7-1, SfREL-His, or P7-1 and SfREL-His, as well as recombinant bacmids expressing Ub-HA and MyD88-Flag. Alternatively, the Sf9 cells were treated with MG132 and detected, as described above. Additionally, viruliferous *S*. *furcifera* insects were microinjected with 10 μM MG132 or DMSO. At 2 days post microinjection, the insects were tested for the accumulation of MyD88 in western blot assay using MyD88 antibody. Insect GAPDH served as a reference protein. The band intensities of proteins were measured using ImageJ software (https://imagej.nih.gov/ij/).

### Sequence homology analysis

The amino acid sequence encoded by *SfREL* was predicted using the online software Smart (http://smart.embl-heidelberg.de/). The amino acid sequences of SfREL and eight vertebrate RING E3s, including RNF114 (XP_034269693.1), RNF125 (XP_005337512.1), RNF138 (XP_016287034.1), RNF166 (XP_029376384.1), TRIM21 (XP_003515416.2), TRIM25 (XP_007622305.1), TRIM27 (AAH69924.1), and TRIM38 (XP_027265051.1), were aligned using the MAFFT server (https://mafft.cbrc.jp/alignment/) in the Geneious 5.0 software. Additionally, the RING domains of the RING E3 homologs from six insect species (*S*. *furcifera*, *Laodelphax striatellus*, *Nilaparvata lugens*, *Recilia dorsalis*, *Nephotettix cincticeps*, and *Spodoptera frugiperda*) were obtained from transcriptome data and subjected to multiple full-length protein sequence alignment followed by maximum likelihood (ML) analysis using MEGA7 software. Furthermore, the amino acid sequences of defensin from seven insect species (*S*. *furcifera*, *N*. *lugens*, *Formica aquilonia*, *Aedes aegypti*, *Drosophila melanogaster*, *Protophormia terraenovae*, and *Stomoxys calcitrans*) were obtained from GenBank and subjected to multiple full-length protein sequence alignment followed by maximum likelihood (ML) analysis using MEGA7 software. The number of Toll receptors, MyD88 and Dorsal in Toll pathway and AMPs of *S*. *furcifera*, *Drosophila melanogaster*, and *Bombyx mori* were counted based on NCBI data and previous reports [32,57,58].

### Statistical analyses

All the data were analyzed using GraphPad Prism version 8.0. Mean values were compared among groups using a student *t*-test and Tukey’s honest significant difference (HSD) tests. The data were back-transformed after analysis for presentation in the text and figures.

## Supporting information

S1 Fig Sequence alignments of defensin homologs from *S*. *furcifera* and other species. (A) The number of Toll receptors, MyD88 and Dorsal in Toll pathways and AMPs of *S*. *furcifera*, *Drosophila melanogaster*, and *Bombyx mori* was counted based on NCBI data. (B) Sequence alignments of the defensin homologs from *S*. *furcifera*, *Nilaparvata lugens*, *Aedes aegypti*, *Drosophila melanogaster*, *Formica aquilonia*, *Protophormia terraenovae* and *Stomoxys calcitrans*. The defensins contain conserved DEFL defensin-like domain at its C-terminus.
(TIF)

S2 Fig Sequence alignments of RING domains of E3 ubiquitin ligase homologs from *S*. *furcifera* and other species. (A) Amino acid sequence alignments of the RING domains among SfREL and eight vertebrate species. (B) Amino acid sequence alignments of the RING domains among E3 ubiquitin ligase homologs from *S*. *furcifera* and 5 insect species (*Laodelphax striatellus*, *Nilaparvata lugens*, *R*. *dorsalis*, *Nephotettix cincticeps* and *Spodoptera frugiperda*). The RING domains contain seven conserved cysteine residues and one histidine residue, and these residues coordinate two zinc ions. (C) An E3 ligase mutant SfREL (H61Y) in which His-61 was replaced by Tyr in the C3HC4-type RING domain.
(TIF)

S3 Fig Ubiquitination of GST-MyD88 by SfREL-HA was analyzed using GST antibody. (TIF)

S1 Table Primers used in this study. (XLS)
