# Supplementary figures and images for: Arboviruses antagonize insect Toll antiviral immune signaling to facilitate the coexistence of viruses with their vectors
Source: PLoS Pathog. 2024 Jun 12;20(6):e1012318. doi: 10.1371/journal.ppat.1012318 (PMC11198909; doi:10.1371/journal.ppat.1012318)

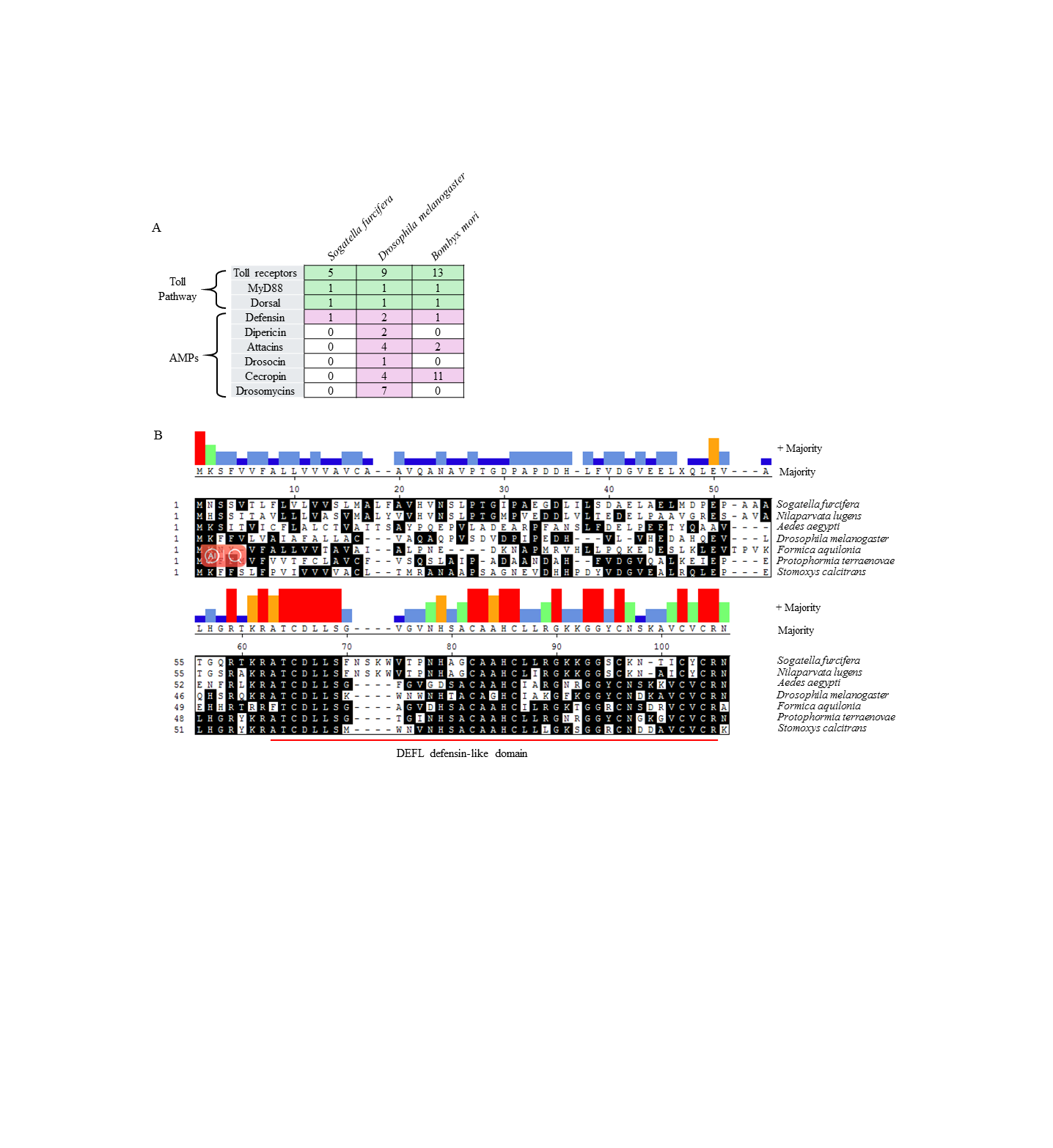

Supplement: S1 Fig — (A) The number of Toll receptors, MyD88 and Dorsal in Toll pathways and AMPs of S. furcifera, Drosophila melanogaster, and Bombyx mori was counted based on NCBI data. (B) Sequence alignments of the defensin homologs from S. furcifera, Nilaparvata lugens, Aedes aegypti, Drosophila melanogaster, Formica aquilonia, Protophormia terraenovae and Stomoxys calcitrans. The defensins contain conserved DEFL defensin-like domain at its C-terminus. (TIF) [file ppat.1012318.s001.TIF]

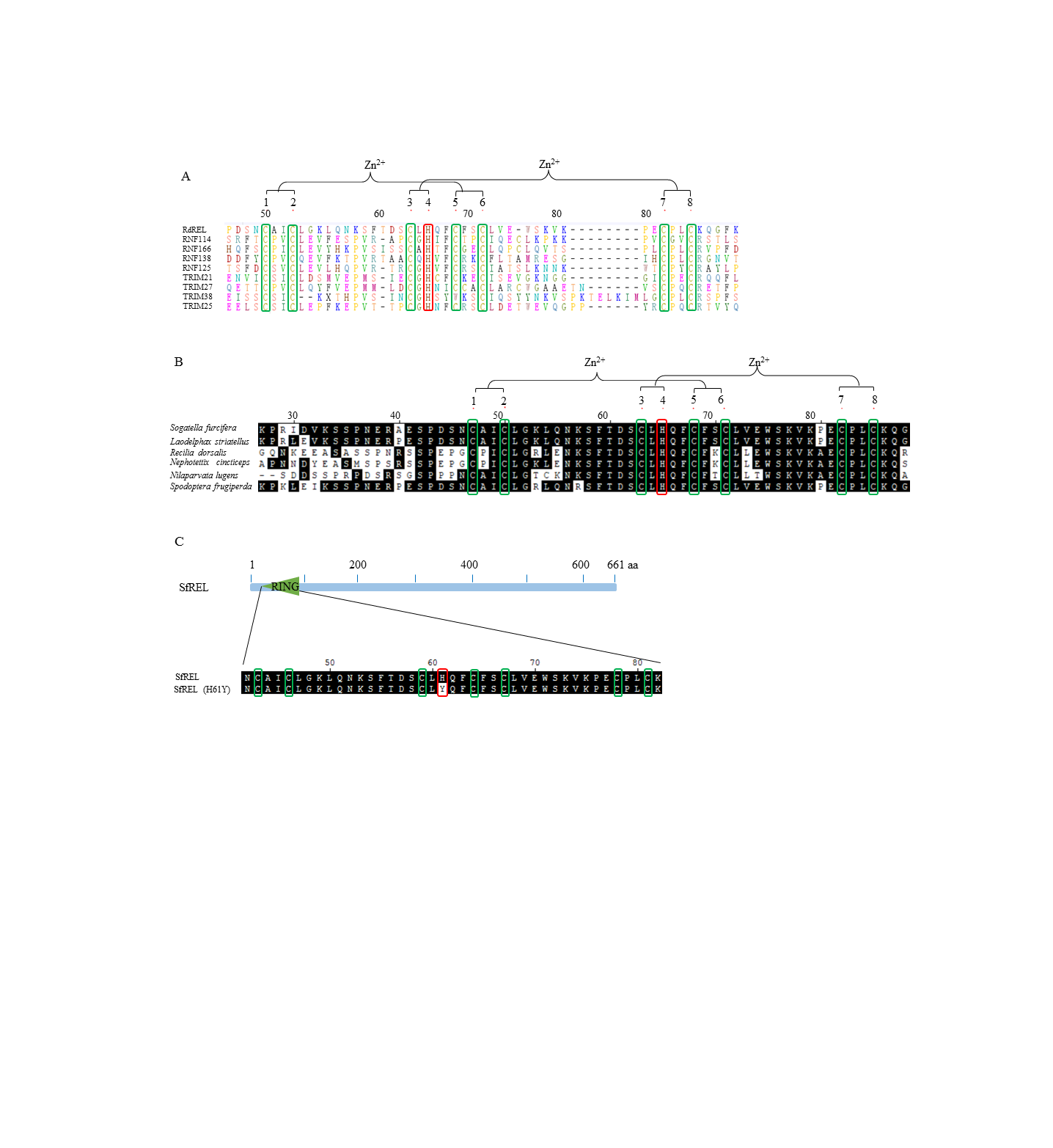

Supplement: S2 Fig — (A) Amino acid sequence alignments of the RING domains among SfREL and eight vertebrate species. (B) Amino acid sequence alignments of the RING domains among E3 ubiquitin ligase homologs from S. furcifera and 5 insect species (Laodelphax striatellus, Nilaparvata lugens, R. dorsalis, Nephotettix cincticeps and Spodoptera frugiperda). The RING domains contain seven conserved cysteine residues and one histidine residue, and these residues coordinate two zinc ions. (C) An E3 ligase mutant SfREL (H61Y) in which His-61 was replaced by Tyr in the C3HC4-type RING domain. (TIF) [file ppat.1012318.s002.TIF]

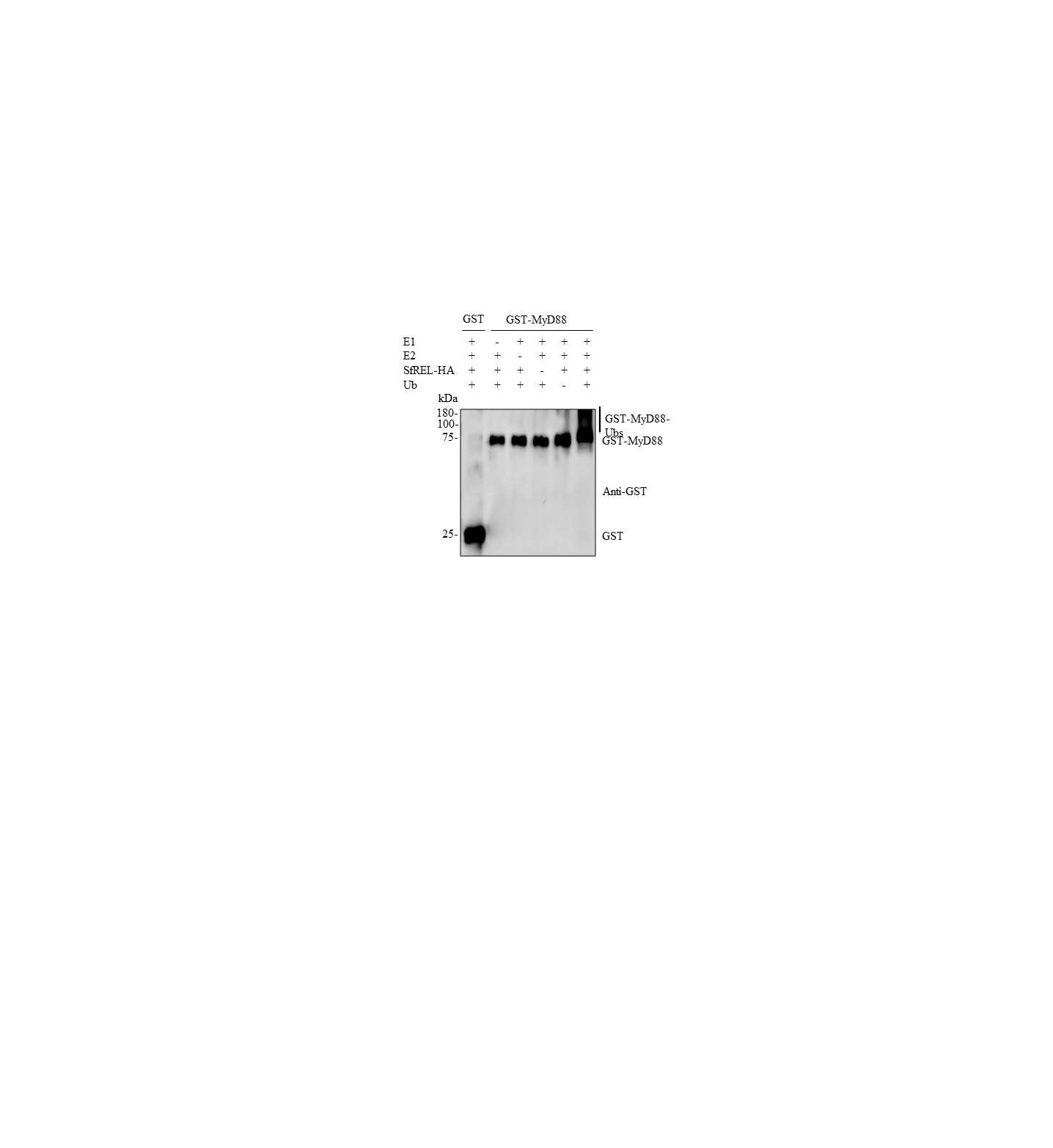

Supplement: S3 Fig — (TIF) [file ppat.1012318.s003.TIF]
